# Sweetness induces sleep through gustatory signalling independent of nutritional value in a starved fruit fly

**DOI:** 10.1038/s41598-017-14608-1

**Published:** 2017-10-30

**Authors:** Tatsuya Hasegawa, Jun Tomita, Rina Hashimoto, Taro Ueno, Shoen Kume, Kazuhiko Kume

**Affiliations:** 1 Department of Neuropharmacology, Nagoya City University, Nagoya, Japan; 2 Department of Stem Cell Biology, Institute of Molecular Embryology and Genetics, Kumamoto University, Kumamoto, Japan; 3 Present Address: Toho University, Faculty of Science, Tokyo, Japan; 4 Present Address: Tokyo Institute of Technology, Yokohama, Japan

## Abstract

Starvation reduces sleep in various animal species including humans and fruit flies. Immediate hunger and the following insufficient nutritional status resulting from starvation may affect sleep and arousal differently. In order to clarify the mechanism underlying the relationship between diet and sleep, we analysed the sleep behaviour of *Drosophila melanogaster* that were either starved or fed with different types of sugars. Starved flies showed longer activity bouts, short sleep bouts and a decreased arousal threshold. Non-nutritive sweeteners such as sucralose and arabinose, which are sweet but not nutritive, induced sleep in starved flies, but sleep bout length and the arousal threshold was short and decreased, respectively. On the other hand, sorbitol, which is not sweet but nutritive, did not induce sleep, but slightly increased the lowered arousal threshold. Activation of sweetness receptor expressing neurons induced sleep in starved flies. These results suggest that sweetness alone is sufficient to induce sleep in starved flies and that the nutritional status affects sleep homeostasis by decreasing the arousal threshold, which resulted in short sleep bouts in *Drosophila*.

## Introduction

The dietary regulation of sleep is a universal phenomenon that occurs in a variety of animals. In human, sleep and metabolism influence each other reciprocally through several endocrine hormones, such as growth hormone, somatostatin, and insulin. For example, it is well established sleep increase growth hormone concentration, while growth hormone deficiency impaired sleep^1,2^. It is clinically remarkable that the disturbance of sleep and circadian rhythm emerged as a novel risk for obesity and diabetes^3–6^. Recent study by Kitamura *et al*. showed one week of recovery from daily potential sleep debt significantly changed the serum hormone levels^7^. Besides the chronic association of metabolism and sleep, we are interested in the effects of acute fasting on sleep. When animals are full, they can sleep without worrying about finding food, but when they are starved, they need to find food immediately. Thus, it is plausible that starvation decreases sleep. *Drosophila melanogaster* has been used as a model animal for sleep studies since 2000 and many sleep regulating genes have been shown to be conserved across the species^8,9^. We first identified the dopamine transporter gene as an arousal regulator in *Drosophila* and analysed the dopamine pathway regulating sleep and arousal^10–14^. In both *Drosophila* and mammals, sleep is related to memory and among the sleep-regulating genes we identified, the following may all function in both sleep and memory regulation: the Fragile X syndrome gene, the NMDA glutamate receptor and calcineurin and its regulator^15–18^. We also examined the relationship between sleep and metabolism and found that c-jun N terminal kinase and recently, Sik3 function to regulate sleep^19,20^. Accordingly, sleep in *Drosophila* is regulated by dietary condition. Keene *et al*. found that starvation decreased sleep, which is reversed by sucrose but not by the non-nutritive sweetener sucralose^21^. The acute sleep decrease by starvation was enhanced by the inhibition of clock neurons and did not require the mushroom body. Catterson *et al*. reported sexual differences and the involvement of protein in the regulation of sleep^22^. Linford *et al*. reported the changes in dietary composition modulated the architecture of sleep rather than the amount of sleep^23^. In contrast, we reported that a high calorie diet also decreases sleep^24^.

Sugars have both sweet taste and nutritional value, and naturally occurring sugars usually possess both. However, some of the sugars and non-nutritive sweeteners are sweet but not nutritive while other sugars are not sweet but nutritive. Flies can assess sweetness and nutritional value separately and they differentially react to them^25–27^. They sense sweetness through gustatory taste receptors^28–30^. Nutritional value is also sensed through other types of gustatory receptors^31,32^. Linford *et al*. reported that, in addition to nutritional (metabolic) value, gustatory perception is required for behavioural changes^33^.

In this study, we examined how sweetness and nutrient value regulate sleep and wake behaviour differently in *Drosophila melanogaster*.

## Materials and Methods

### Fly stocks and maintenance

Flies (*Drosophila melanogaster*) were reared on a standard corn meal, yeast and glucose agar medium at 24.5 °C under a 12-h:12-h light: dark (LD) cycle as described before unless otherwise stated^10^. Control (*w* ^1118^) and *UAS-TrpA1* fly stocks were described previously^10,13^. *Gr5a-GAL4* (#57592), *Gr43a-GAL4* (#57636) and *Gr64a-GAL4* (#57661) were obtained from the Bloomington Stock Center; *Cha-GAL80*, *Gad-GAL80* and *UAS-Shi* ^*ts1*^ were from Dr. T. Sakai (Tokyo Metropolitan University). To remove possible modifiers and allow for comparisons in a common genetic background, we outcrossed all the strains into a control (*w* ^1118^) background for at least five consecutive generations.

### Locomotor activity and sleep analysis with starvation or sugar food

Male 2-7-d-old flies were used. They were reared for a minimum of 24 hrs on 150 mM sucrose, 1% agar food before starting the starvation assay. For the arousal threshold and food consumption assay, they were reared in vials containing ordinary food and used directly. For locomotor activity and sleep analysis, flies were placed individually in glass tubes (length, 65 mm; inside diameter, 3 mm) with 1% agar and 150 mM sucrose on one end at 24.5 °C. They were entrained for 1 d (Pre) to LD cycle. At ZT 0 (lights-on) of day 1, they were transferred to glass tubes with the specified test medium (1% agar with or without additions). Locomotor activity was monitored by recording infrared beam crossings by individual flies in 1 min bins using the *Drosophila* activity monitoring system (Trikinetics, MA, USA). On the basis of previous reports, sleep was defined as periods of inactivity lasting 5 min or longer. Daily sleep time was calculated with original software written in Excel or R 3.2.2. We defined the continuous disappearance of infrared beam crossings as a fly’s death and excluded the data of dead flies after their death.

### Arousal threshold assay

To examine responsiveness to stimuli during sleep, we devised a customized instrumental setup to provide air and odour stimulation. We connected a *Drosophila* Activity Monitor 2 equipped with a MAN2 gas distribution manifold (Trikinetics) to two airflow meters and bottles of water and an odour source (3-octanol, Sigma, USA) in parallel to an air pump (Anest-Iwata FX2047 air compressor, Japan). We drilled small holes (diameter, 0.7 mm) on plastic tubes (length, 65 mm; inside diameter, 3 mm) at 13 mm from one end as the escape route for the airflow. The agar food was filled to the ends of the tubes near the holes and capped. After loading the flies, the other ends were connected to a gas manifold. As schematized in Sup. Figure 1, we placed 2-7-d-old male flies, which were cultured on ordinary food, individually into tubes containing sugar food at ZT 10 on day 1. Flies were kept in the tubes for 29 to 33 hrs and at ZT 15-19 on day 2, two types of stimuli were applied. First, airflow (1 L/min) that was passed through water without odour was delivered to the gas manifold and was maintained for 30 min. Then a half of the airflow (0.5 L/min) was replaced with another airflow that was passed through a bottle of 3-octanol.

After the assay, we determined the number of flies that awakened, i.e. started to move, within 5 min after stimulation from the number of flies sleeping, i.e. not moving, during 5 min before stimulation. In order to subtract spontaneous waking, we also counted the number of flies which awakened within 5 min before stimulation among the flies sleeping during 10 to 5 min before stimulation. Then we defined the arousal response rate (%) as follows; Response (%) = ((The number of flies awakened in the 5 minutes after stimulation/The number of flies sleeping during the 5 min before stimulation) − (The number of flies waking in the 5 minutes before stimulation/The number of flies sleeping 10 to 5 min before stimulation)) × 100.

### Neuronal regulation with *dTrpA1* or temperature sensitive *Shibire*

*Gr-GAL4* driver lines with or without *UAS-dTrpA1* or *UAS-shi (shibire)* ^*ts1*^ were reared at 22 °C. They were placed individually in glass tubes with 150 mM sucrose food on day 0 at 22 °C and then transferred to a tube with different sugar food at ZT 0 on day 1. The temperature was shifted from 22 °C to 29 °C at ZT 0 on day 2.

### Statistical analysis

In all the bar graphs, data are represented as mean ± s.e.m. Quantitative data of multi-group datasets were analysed by a one-way ANOVA with a Tukey-Kramer HSD post hoc test as described in the figure legends using Excel or R 3.2.2. We excluded the data from a fly if the infrared beam crossings had ceased during the analysis period.

## Results

### Starvation induced sleep decrease was inverted by non-nutritive sweeteners

**Figure 1:**
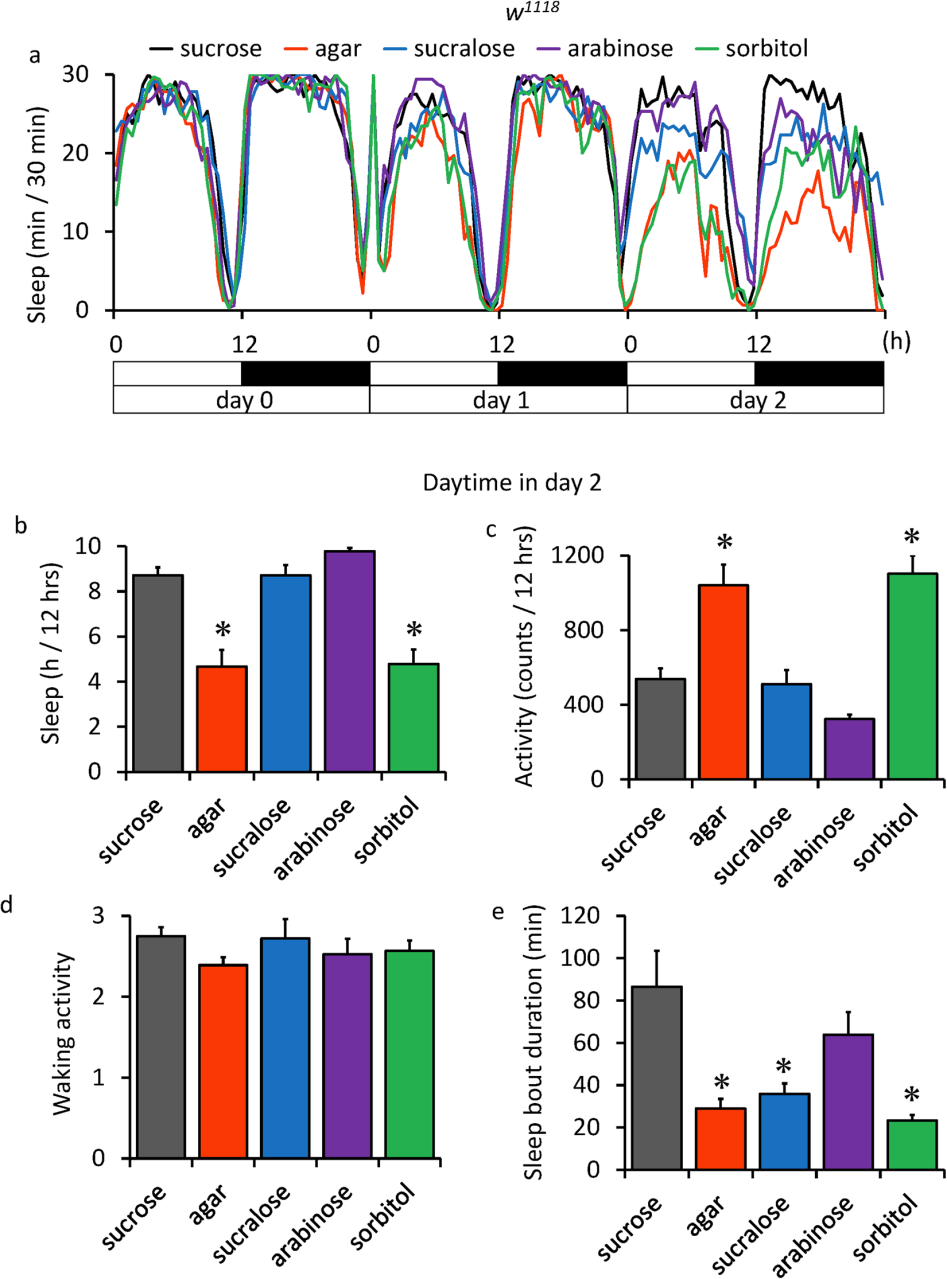
Sweeteners induced sleep in starved flies. Flies fed with 150 mM sucrose on day 0 were transferred to agar containing 150 mM sucrose, no sugar, 150 mM sucralose, 300 mM arabinose or 300 mM sorbitol on day 1, and locomotor activity was monitored for 3 d in the 12-h light: dark cycle (LD) condition. (**a**) Average sleep amounts in 30-min intervals for each group having one of five foods on day 1 and day 2 are plotted (n = 16, 15, 14, 14, 14 flies, respectively). Day and night are depicted by the white and black bars, respectively. (**b-e**) Summary of the sleep parameters of daytime (ZT 0–12) of day 2. Total sleep amount (**b**), total activity counts (**c**), waking activity index (**d**) and mean sleep bout duration (**e**) are shown. Waking activity is defined as the total daily activity divided by the length of the daily active period. Data are represented as mean ± s.e.m. Statistical significance between sucrose and each food was calculated using a one-way ANOVA with a Tukey-Kramer HSD post hoc test. *P < 0.05.

On day 0, flies were kept in tubes with 150 mM (ca. 5%) sucrose/1% agar food and then on day 1 they were transferred to another tube with 1% agar food containing different sugars. As shown in Fig. 1a, the sleep amount of control flies fed with sucrose (black) did not change for three days. Starved flies fed with agar and water (red) started to show a reduction in sleep on day 2, 24 hrs after transfer. Flies fed with either 150 mM (ca. 5%) sucralose (blue) or 300 mM (ca. 5%) arabinose (purple), which are sweet but have no nutrient value to flies, had a higher level of sleep compared to starved flies. Flies fed with 300 mM (ca. 5%) sorbitol (green), which is nutritive but not sweet, showed reduced sleep, almost equivalent to starved flies.

Figure 1b to e show the quantification of sleep parameters during the 12 hrs light period on day 2, the second day after the transfer to different sugar foods. The amount of total sleep (Fig. 1b) decreased to approximately half and total activity counts (Fig. 1c) increased - approximately twice in starved flies than in control sucrose-fed flies. Both sucralose and arabinose almost completely reversed the change in sleep by starvation while sorbitol had a very small effect. The waking activity index did not change significantly between different sugar diets, indicating the locomotor activities during wakefulness were not affected by starvation (Fig. 1d). The average length of one sleep bout was shortened in starved flies and in sucralose and sorbitol-fed flies (Fig. 1e). Therefore, the number of activity-rest cycles, which indicate how many times a fly sleeps during a 12-hour period, increased in sucralose and arabinose-fed flies compared with the control (not shown). The average length of one activity bout was prolonged significantly in starved flies and sorbitol-fed flies, but was shortened in sucralose and arabinose-fed flies (not shown). The results indicate that sucralose reduced the length of both one activity bout and one sleep bout compared with sucrose, but in sum, they did not change total activity and total sleep amount. Sorbitol-fed flies did not change total activity and total sleep compared with starved flies, and the length of one activity bout and sleep bout were also comparable to starved flies. These results were most evident during the daytime in day 2, but similar sleep changes were significant during the daytime in day1 and the nighttime in day2 (Sup. Figure 2).

We confirmed that the difference between these sugars was dependent on their sweetness and nutritive value by following supplementary experiments. First, we measured survival time of the flies on these sugars. Sucralose and arabinose did not have any effects and the survival times on these sugars were equivalent to that on agar and water only food (Sup. Figure 3a). Sorbitol alone extended the survival time, but some of the flies started to die after 3 days. When sorbitol was mixed with arabinose, survival was similar to flies on sucrose. Next, we measured the amount of food consumed by the flies. All the sugars were ingested by flies; sucrose was most effective and sorbitol was least effective (Sup. Figure 3b).

Since a previous study by Keene *et al*. (Keene *et al*., 2010) reported 1 mM sucralose did not affect starvation-induced sleep decrease, we examined the effects of various concentrations of sucralose and arabinose. Concentrations of less than 6.25 mM for sucralose and 12.5 mM for arabinose did not result in increased sleep, but higher concentrations (25 mM or 50 mM, respectively) were effective (Sup. Fig. 4ab). Thus, our results did not conflict with their results. The effect of sorbitol is intermediate. Since it does not taste sweet, flies do not eat sorbitol as much as sucrose.

### Arousal threshold was lowered by starvation, and was not affected by non-nutritive sweeteners

**Figure 2:**
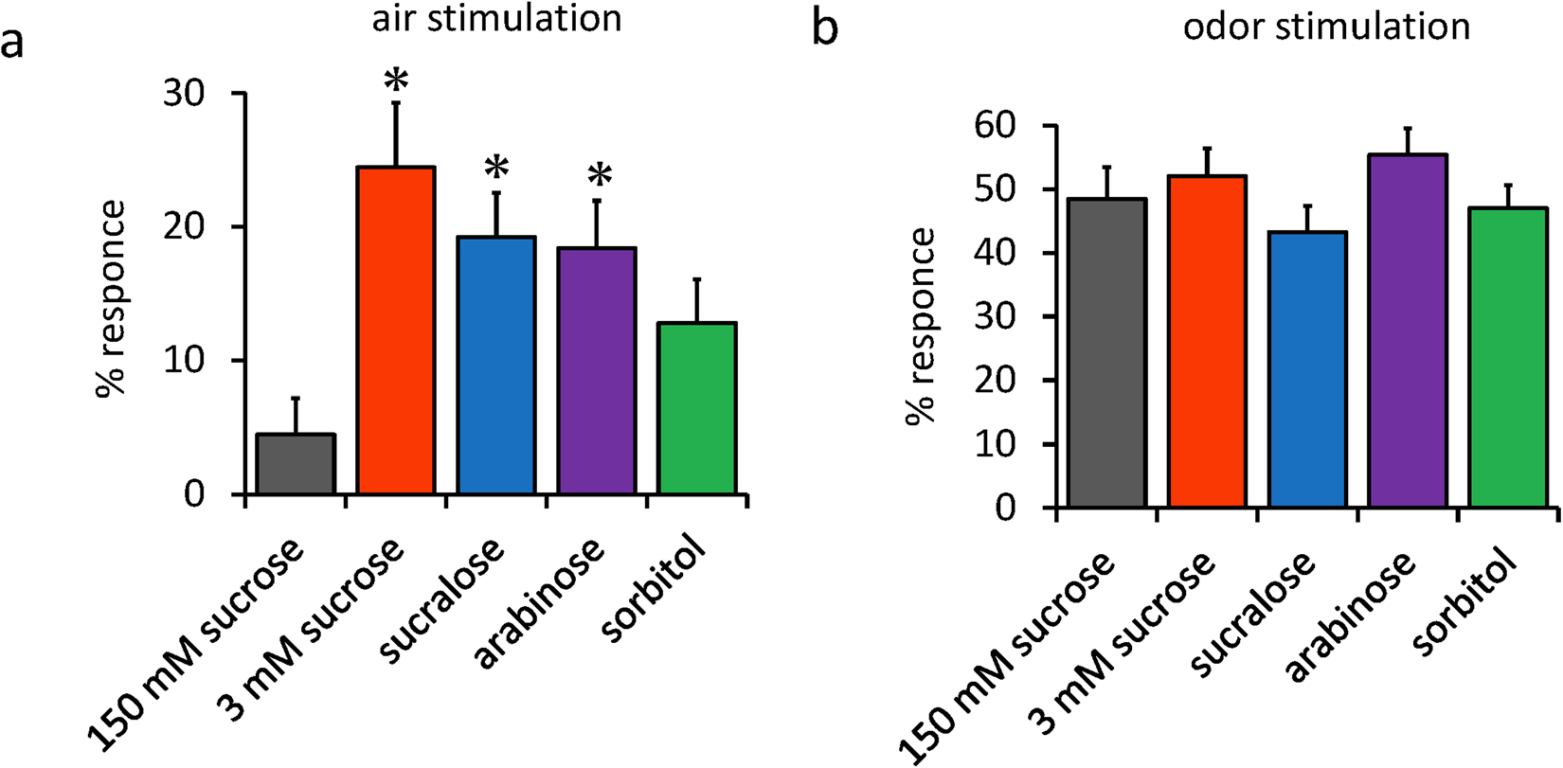
Arousal threshold was lowered by starvation and was not affected by non-nutritive sweeteners. Flies fed with different foods in activity monitor tubes were given (**a**) a weak airflow stimulation followed by (**b**) an airflow containing 3-octanol odour. Locomotor activities were continuously monitored and the rate of flies that changed their states from sleep to awake within 5 min after the stimuli were determined as described in the Materials and Methods. In order to subtract the background waking rate independent of stimuli, the rate of flies that spontaneously changed their state from sleep to awake within the 5-min period before stimuli were calculated and subtracted from the above value. One experiment was done using a group of 32 flies with one food condition and the minimum number of flies included for the calculation was 21. The number of experiments for each food condition was 9, 9, 9, 10, and 10, respectively. Data are represented as mean ± s.e.m. Statistical significance between 150 mM sucrose and each food was calculated using a one-way ANOVA with a Tukey-Kramer HSD post hoc test. *P < 0.05.

We next measured the arousal threshold with a weak air flow stimulation and a successive odour stimulation as shown schematically in Sup. Figure 1. In this assay, we used 3 mM sucrose instead of no sugar, because it requires a significant number of flies sleeping. Complete starvation makes flies sleep for very short period, which would make it difficult to perform this assay. In a prior experiment, we tested various concentrations of sucrose and confirmed that 3 mM sucrose is enough to induce starvation-induced sleep loss (Sup. Figure 1b). As shown in Fig. 2a, starved flies fed with 3 mM sucrose responded to the weaker stimulation more sensitively. More than 25% of starved flies responded to the air flow stimulation, to which less than 5% of the satiated flies fed with 150 mM sucrose responded. The responses of flies fed with sucralose and arabinose were significantly higher than satiated flies, while that of flies fed with sorbitol was higher but the difference was not significant compared with satiated flies. For the odour stimulation, which induced a 50% response in the satiated flies, there were no significant differences between food conditions.

These results suggest that the lowering effects of starvation on the arousal threshold from the sleep state are not affected by non-nutritive sweeteners.

### Activation of gustatory sugar receptor expressing neurons inverted the starvation induced sleep decrease

**Figure 3:**
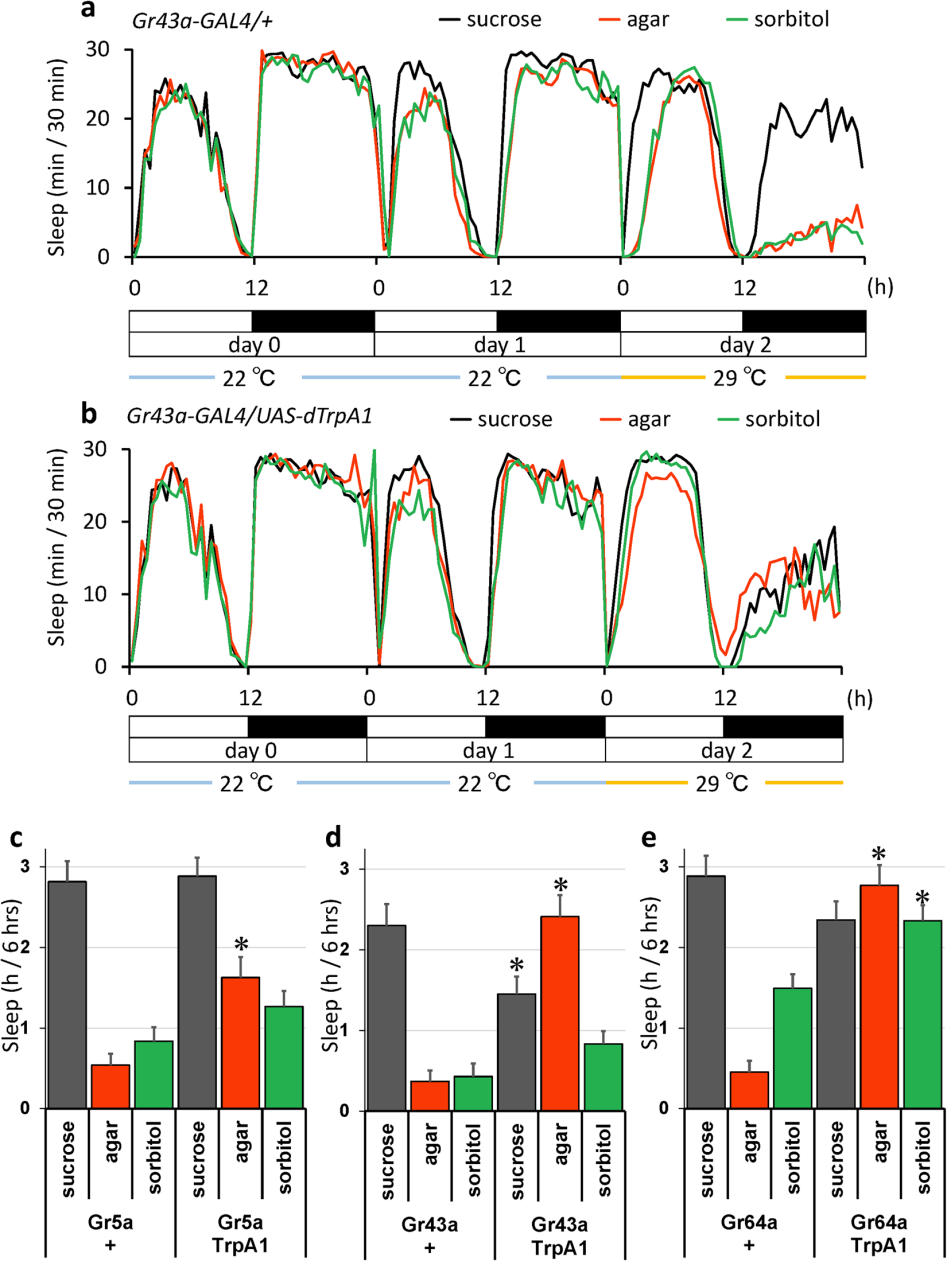
Activation of gustatory sugar receptor expressing neurons in starved flies induced sleep. Flies of indicated genotypes fed with 150 mM sucrose at 22 °C on day 0 are transferred to different foods as in Fig. 1 at 22 °C on day 1, and the temperature was shifted to 29 °C at ZT 0 on day 2 to activate dTrpA1 expressing gustatory neurons. (**a** and **b**) Average sleep amounts in 30-min intervals of each group are plotted. (**a**) *Gr43a-GAL4/*+ (n = 32, 30, 31) (**b**) *Gr43a-GAL4/UAS-dTrpA1* (n = 32, 30, 32) (**c**–**e**) Total sleep amount for control and flies with Gr neuron activation on the first half of nighttime (6 hrs from ZT 12 to ZT 18) on day 2; *Gr5a* (n = 19, 16, 18, 32, 16, 32), *Gr43a* (n = 32, 18, 29, 32, 19, 32), *Gr64a* (n = 20, 13, 19, 27, 15, 29). Data are represented as mean ± s.e.m. Statistical significance between control and flies with Gr expressing neuronal activation was calculated using a one-way ANOVA with a Tukey-Kramer HSD post hoc test. *P < 0.05.

In order to confirm that the sweet taste is capable of inducing sleep, we activated neurons expressing gustatory sugar receptors using the GAL4-UAS system and a heat-activatable cation channel dTrpA1. We expressed dTrpA1 using *Gr43a*, *Gr5a* and *Gr64a-GAL4* combined with *UAS-dTrpA1* and examined sleep amount before and after heat activation of dTrpA1 under starved and sugar-fed conditions. The control line, *Gr43a-GAL4* driver alone did not cause any changes at high temperature, and agar (red line) and sorbitol-fed flies (green line) showed a reduction in sleep compared to the control (black line) during the night of day 2 (Fig. 3a). However, the activation of Gr43a expressing neurons induced sleep under starvation, and even agar or sorbitol fed flies demonstrated sleep almost equivalent to the control (Fig. 3b). Similarly, the activation of Gr5a and Gr64a expressing neurons induced sleep under starvation (Sup. Fig. 5). Since flies started to die at high temperature toward the end of day 2, we quantified sleep during the first half of the night (ZT 12–18) in day2. The results shown in Fig. 3c–e suggested that among the three Gr’s, the activation of *Gr43a* or *Gr64a* expressing neurons were as potent as sucrose in the induction of sleep, while activation of *Gr5a* expressing neurons showed weaker effects. The quantifications of sleep were also performed during daytime (ZT0–12) and the second half of the night (ZT18–24) and shown in Sup. Fig. 7a–c and d–f, respectively. The tendency of the results was similar.

We also examined which types of neurons are important by combining the *Gr-GAL4* driver with different types of *GAL80* drivers, which suppress GAL4 signalling. When *Gr-GAL4* was combined with choline-acetyltransferase, *Cha-GAL80*, which suppress GAL4 only in cholinergic neurons, sleep inducing effects of the *Gr43a and Gr64a-GAL4* drivers were abolished almost completely as shown in Sup. Figs 8 and 9. The results indicated that Gr expressing cholinergic neurons are important for the induction of sleep by non-nutritive sweeteners and that the activation of sweet sensor neurons can induce sleep.

### Inhibition of gustatory sugar receptor expressing neurons suppressed sweetener-induced sleep

**Figure 4:**
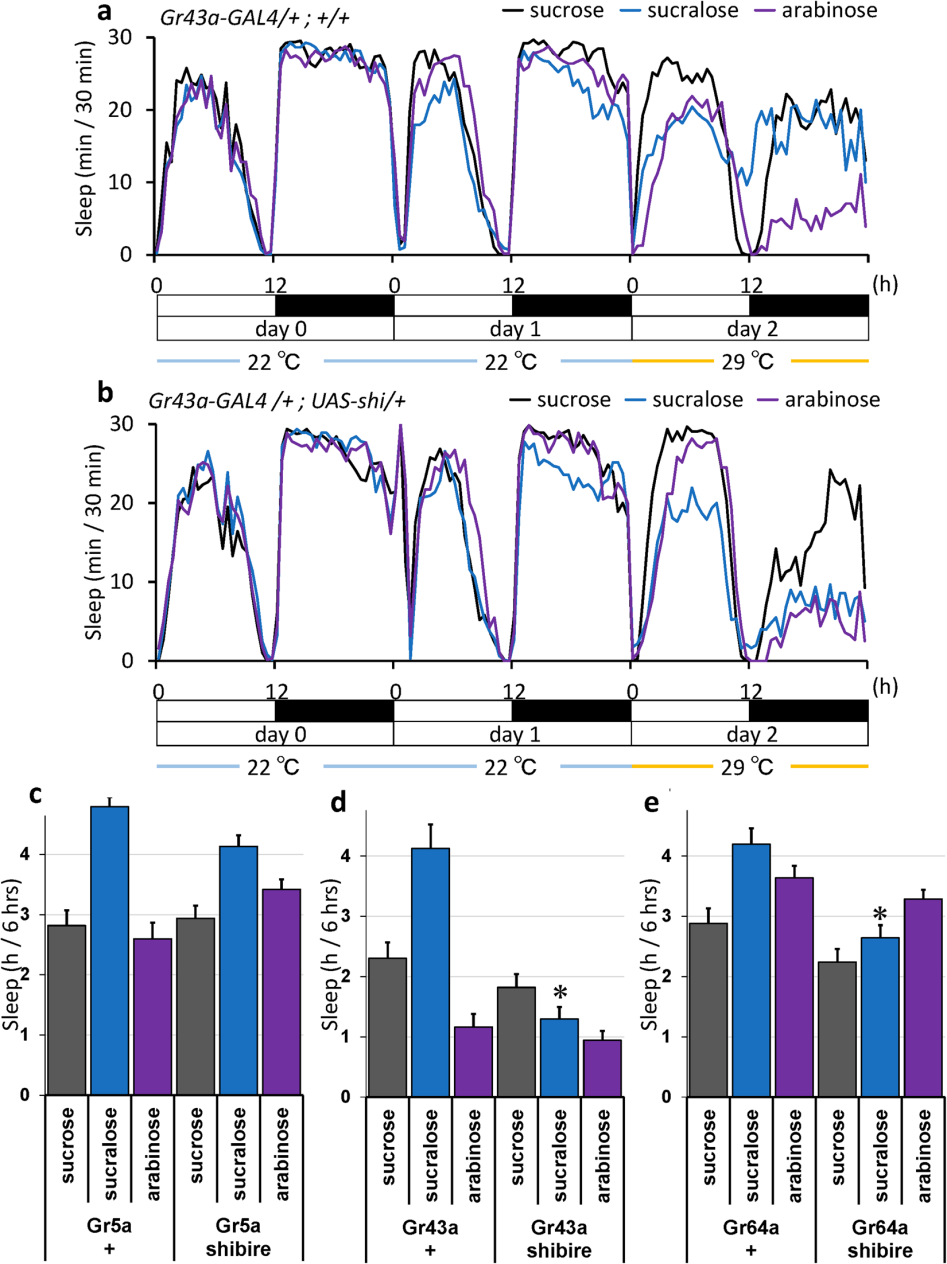
Inhibition of gustatory sugar receptor expressing neurons suppressed sweetener-induced sleep. Flies of indicated genotypes fed with 150 mM sucrose at 22 °C on day 0 were transferred to different foods as in Fig. 1 at 22 °C on day 1, and the temperature was shifted to 29 °C on day 2 to inhibit *Shi* ^ts^ expressing gustatory neurons. (**a** and **b**) Average sleep amounts in 30-min intervals for each group are plotted. (**a**) *Gr43a-GAL4/*+ (n = 32, 32, 32 flies) (**b**) *Gr43a-GAL4/UAS-shi* (n = 32, 32, 32 flies) (**c**–**e**) Total sleep amount for control and flies with Gr neuron inhibition on the first half of nighttime (6 hrs from ZT 12 to ZT 18) on day 2; *Gr5a* (n = 19, 21, 26, 29, 27, 29 flies), *Gr43a* (n = 32, 11, 15, 32, 19, 26 flies) and *Gr64a* (n = 20, 19, 19, 32, 26, 27 flies). Data are represented as mean ± s.e.m. Statistical significance between control and flies with Gr neuron inhibition was calculated using a one-way ANOVA with a Tukey-Kramer HSD post hoc test. *P < 0.05.

Next, we used *UAS-shi (shibire)* ^*ts1*^ in these neurons to express a temperature sensitive dynamin and inhibit synaptic transmission at high temperature where it acts in a dominantly negative manner. As shown in Fig. 4a, the control line/ *Gr43a-GAL4* driver alone showed a similar level of sleep when the flies were fed with sucrose (black line) and sucralose (blue line), but they slept less with arabinose (purple) at high temperature. We do not know why they respond differently to sucralose and arabinose only at high temperature, but it is possible that the difference in sweetness may have affected their responses, since sucralose is much sweeter than arabinose. The inhibition of *Gr43a-GAL4* expressing neurons decreased sleep in sucrose and sucralose fed flies as shown in Fig. 4b. Similarly, the inhibition of Gr5a and Gr64a expressing neurons also decreased sleep as shown in Sup. Figs 10 and 11. The inhibition of neurons expressing either of the three sugar receptors partially suppressed sucrose or sweetener induced sleep. We quantified sleep during the first half of the night (ZT 12–18) in day2 as in Fig. 3. The results showed, among the three, the inhibition of *Gr43a* expressing neurons was the most effective as shown in Fig. 4c–e. As a control experiment, we confirmed that the Gr driver alone did not cause changes.

## Discussion

As we showed in this study, high concentrations of non-nutritive sweeteners induce sleep in starved flies. Previous studies by Keene *et al*. reported that a low concentration of sucralose (1 mM) could not drive sleep^21^. We reproduced their results and found that a higher concentration is required for sleep (Sup. Figure 4). Although 1 mM sucralose was sufficient to induce proboscis extension responses (PER) in flies, it was not enough to induce sleep. We do not know the exact mechanism for the difference, but the temporary characteristics of the two observations are quite different. PER is an assay completed over a very short time such as a few seconds, whereas sleep observation requires a few days. We suspect sweetness sensors need to be persistently activated for maintaining sleep. High concentration non-nutritive sweeteners can be ingested by flies and stay inside their body for a while to activate sweet sensor neurons. Since they are diluted when ingested, high concentrations are required for a prolonged sweetness response. In our study, starvation induced sleep loss but the waking activity was not altered (Fig. 1d), which is different from previous work such as Murakami *et al*., which described the increase in waking activity by starvation^34^. We do not regard the difference is critical since the experimental conditions are quite different such as fly stain, sex and the food condition. While we were preparing this manuscript, Wang *et al*. published an interesting finding that the addition of sucralose to the conventional diet promoted food intake and hyperactivity and decreased sleep^35^. They used 2.5% sucralose (63 mM) with 5.4% sucrose (ca. 160 mM), and food intake began to increase 3 to 4 days after sucralose feeding. They also found NPF plays a critical role. Although the experimental conditions were different, their results also suggest that higher concentration of sucralose may be required for behavioural changes.

Starvation lengthened one wakefulness bout, shortened one sleep bout and decreased the arousal threshold. This indicates that flies use more time for finding food, and wake up earlier in order to find food. Artificial sweeteners, which are sweet but not nutritive, shortened one wakefulness bout, resulting in an increase in total sleep. But they did not reverse the shortening of one sleep bout and the decrease in arousal threshold. This indicated that the flies may have been deceived by the sweetness and started to sleep, but they wake up early since they are not truly satiated. On the other hand, sorbitol could not induce immediate sleep, but once they started to sleep, the flies slept for longer than when they were hungry.

The different information processing of sweetness and nutrient value in *Drosophila* has been described in various ways. Fujita and Tanimura discovered that flies are capable of learning to associate an odour with sorbitol even though sorbitol did not induce PER^26^. They also showed that the intake amount of sorbitol was more than that of water alone, but was far less than that of glucose. Burke and Waddell also found that arabinose induced short-time associative memory to a level almost equivalent to that of sucrose, but it failed to induce long-term (24 hr) memory^25^. They also showed that the addition of sorbitol, which did not induce long-term memory by itself, to arabinose induced significantly strong long-term memory, which suggested both sweetness and nutrition are important. Both studies indicated that *Drosophila* sense nutritional ingredients independently from sweetness. Recent studies revealed that octopamine and dopamine neurons in the mushroom body (MB) mediate short- and long-term memories^36^. It is suggested that specific PAM cluster neurons induce short-term memory, and PAM-α1 neurons induce long-term memory. Since sleep and memory are closely related, sweet taste information may regulate sleep via dopaminergic neurons in the MB.

Sweetness is perceived by several gustatory taste receptors^37^. Taste receptors are expressed in various organs including the labellum, maxillary palp and legs^38^, and those expressed in the legs are important in immediate choice of tastes^39^. Gr5a, Gr61a, Gr43a and Gr64a-f are important in sweetness sensation^31^, and Gr64a is more sensitive to sucrose than Gr5a^37^. The expression patterns of these neurons are also diverse. Gr5a expressing neurons project mainly to the centre of suboesophageal ganglion (SOG) while Gr43a and Gr64a expressing neurons to the dorsal side of it^38^. Activation of *Gr66a* expressing neurons was sufficient for aversive memory induction^25^. In this study, activation of *Gr43a* and *Gr64a* expressing neurons increased sleep significantly in starved flies. Since the sensitivity of Gr5a and Gr64a is significantly different, different kinds of taste neurons are involved in sleep regulation in flies. When expressed in the brain, *Gr43a* is reported to be a fructose sensor and to function as a nutrient sensor^32^. We identified that cholinergic *Gr43a* expressing neurons were important for mediating sleep, but *Gr43a* expressing neurons in the brain are not cholinergic neurons. Our results suggested that the gustatory taste receptor, Gr43a, has a role in not only nutrition sensing, but also sleep regulation.

Interestingly, the response to the sweet sensation changes depending on the satiety conditions of the host animal. The taste information is transmitted to the second-order neurons which connect primary sensory neurons to the higher brain area^40^. When starved, the animals start to feed and ingest sugars but they do not when they are full. Using *NP1562*-*GAL4*, Kain and Dahanukar identified a positive second order neuron which connects sweet-sensor neurons to the antennal mechanosensory and motor centre (AMMC)^41^. The inhibition of these second order neurons suppressed immediate sweetness-induced PER. Yapici *et al*. identified the second order neuron called IN1, which integrates sweetness information with hunger signals^42^. IN1 connects predominantly with *Gr43a* and *Gr64a* expressing neurons but not with *Gr5a* expressing neurons. This may be comparable to this study, which showed that the inhibition of *Gr43a* and *Gr64a* expressing neurons was more effective than that of *Gr5a* expressing neurons.

Recent studies showed that *Drosophila* insulin-like peptides (DILPs) mutants could reduce sleep and expression of DILP2 was reduced in starved flies^43^. It is also known that the nutrient-responsive hormone, CCHamide-2 (CCHa2), regulates the expression and release of some DILPs^44^. These DILPs, and regulators like CCHa2, may be the downstream of sweet taste information, regulating sleep and nutrient information and mediating arousal threshold.

As a technical issue, previous studies such as Keene *et al*. described that the decrease in sleep by starvation was observed at the first night of the starvation, which is 12 to 24 hr after starvation^21^. The stock we used did not show any changes in sleep and locomotor activity during the first day and night of the starvation, and their sleep started to decrease 24 to 36 hours after starvation. We do not know the exact cause of this 12-hr difference, but since our control flies show lower locomotor activity counts and longer sleep on average than flies from the other lab (data not shown), we suspect they are more resistant to starvation. Another factor may be prior food conditions before starting starvation. We raised the flies on a conventional food and then kept them on 150 mM sucrose for about 1 day, but in previous studies, the flies were kept on conventional food just before the starvation. Due to the characteristics of our control strain, we analysed data from the 2^nd^ starvation day, when the flies show clear effects of starvation.

## Electronic supplementary material

Supplementary Information
