# Supplementary material for: Sweetness induces sleep through gustatory signalling independent of nutritional value in a starved fruit fly
Source: Sci Rep. 2017 Oct 30;7:14355. doi: 10.1038/s41598-017-14608-1 (PMC5662574; doi:10.1038/s41598-017-14608-1)
Supplement: Supplementary file 1 — Supplementary Information [file 41598_2017_14608_MOESM1_ESM.pdf]

Supplementary Information

Sweetness induces sleep through gustatory signalling independent of nutritional value in a starved fruit fly.

Tatsuya Hasegawa[1*], Jun Tomita[1,2*], Rina Hashimoto [2*], Taro Ueno [2,3], Shoen Kume [2,4], Kazuhiko Kume [1,2,5]

1. Department of Neuropharmacology, Nagoya City University
2. Department of Stem Cell Biology, Institute of Molecular Embryology and Genetics, Kumamoto University
3. Present address: Toho University, Faculty of Science
4. Present address: Tokyo Institute of Technology
5. Corresponding author: kume.kazuhiko@gmail.com,

Supplemental Materials and Methods

Survival time

Locomotor activities of flies were monitored similarly to the sleep assay and the death of a fly was determined by the continuous disappearance of infrared beam crossings.

Multi capillary feeding assay (CAFÉ assay)

To quantify the amount of food intake, we used a MutiCAFÉ assay [1], which is based on the CAFÉ assay [2]. In this assay, 40 male flies were housed in a plastic case (97×67.5×19.2 mm, Sanplatec, Tokyo, Japan), and glass capillaries (length, 32 mm; inside diameter, 0.44 mm, Sanplatec) filled with liquid medium containing 0.125 mg/ml FD&C blue #1 (Kyoritsu Food, Japan) were attached to a microscope slide (26×76 mm, Sanplatec) in the case. We recorded their food intake for 24 h under LD cycle. The rate of image capture was 1 frame per hour and we calculated the amount of liquid medium losses using the Image J9 software. As a control, we prepared a case without flies and determined evaporative losses. We quantified the amount of food intake by eliminating evaporative losses from the liquid medium losses.

Supplemental References

1. Sellier, M.-J., Reeb, P. & Marion-Poll, F. Consumption of bitter alkaloids in Drosophila melanogaster in multiple-choice test conditions. *Chem. Senses* **36,** 323–34 (2011).
2. Ja, W. W. *et al.* Prandiology of Drosophila and the CAFE assay. *Proc. Natl. Acad. Sci. U. S. A.* **104,** 8253–6 (2007).

Supplemental Figure Legends

Sup. Fig. 1. Arousal threshold assay

(a) Schematic representation of experimental procedure. At ZT 10 on day 1, flies were directly placed in activity monitor tubes with different sugar foods as described in the Materials and Methods. Stimuli were applied to flies between 29-33 h (ZT 15-19) after. The first stimulus was an airflow (1 L/min) without an odor and the second was an airflow with a 3-octanol odor. After giving an odor airflow, we examined the rate of flies shifting from sleep to awake in a 5 min period.
(b) Dose dependency of sleep change by sucrose. On day 1, flies fed on ordinary food were directly placed in tubes with agar containing indicated concentrations of sucrose, and locomotor activities were monitored. Average sleep amounts in 30-min intervals for each group were plotted (n = 16).

Sup. Fig. 2. Sleep quantification supplemented to Fig 1c-e.

As in Fig. 1, total sleep amount and total activity counts of daytime (ZT 0-12) of day 1 (a, b), nighttime (ZT 12-24) of day 1 (c, d) and nighttime (ZT 12-24) of day 2 (e, f).

Sup. Fig. 3. Survival time and the amount of food intake in flies fed with each type of sugar food.

(a) Flies fed with 150 mM sucrose were transferred to activity monitor tubes with agar containing either 150 mM sucrose, no sugar, 150 mM sucralose, 300 mM arabinose, 300 mM sorbitol, or a mix of 300 mM arabinose and 300 mM sorbitol (n = 16 flies for each food), and locomotor activity was monitored. Times of death were determined as the point from which no activity was recorded and then the survival rate was calculated.

(b) A group of 40 flies fed with 150 mM sucrose for 1 day were transferred to a plastic case with glass capillaries filled either with stained 150 mM sucrose, 150 mM sucralose, 300 mM arabinose, 300 mM sorbitol, or a mix of 300 mM arabinose and 300 mM sorbitol. The food consumption in each box was monitored by the decrease of the food as described in the Supplemental Materials and Methods.

Sup. Fig. 4. Dose dependency of sleep induction by non-nutritive sweeteners

As in Fig 1, flies fed with 150 mM sucrose on day 0 were transferred to agar containing either 150 mM sucrose, no sugar, or indicated concentrations of (a) sucralose or (b) arabinose on day 2, and locomotor activities were monitored. Average sleep amounts in 30-min intervals for each group were plotted (n = 10).

Sup. Fig. 5. Activation of gustatory sugar receptor expressing neurons in starved flies induced sleep.

Same as Fig. 3ab except for the difference of the Gr driver. (a) *Gr5a-GAL4/+* (n = 19, 18, 18) (b) *Gr5a-GAL4/UAS-dTrpA1* (n = 32, 25, 32)

Sup. Fig. 6. Activation of gustatory sugar receptor expressing neurons in starved flies induced sleep.

Same as Fig. 3ab except for the difference of the Gr driver. (a) *Gr64a-GAL4/+* (n = 20, 17, 19) (b) *Gr64a-GAL4/UAS-dTrpA1* (n =27, 23, 29)

Sup. Fig. 7. Sleep quantification supplemented to Fig 3c-e.

Same as Fig 3, total sleep amount and total activity counts of daytime (ZT 0-12) of day 2 (a-c) and the second half of nighttime (ZT 18-24) of day 2.

Sup. Fig. 8. Cholinergic gustatory neurons are important in inducing sleep in starved flies.

Same as Fig. 3, flies of indicated genotypes were transferred to different foods, and the temperature was shifted to 29°C to activate dTrpA1 expressing gustatory neurons except for cholinergic neurons, which express GAL80. (a-d) Average sleep amounts in 30-min intervals for each group are plotted. (a) *Gr43a-GAL4/+;Cha-GAL80/+* (n = 32, 32, 32) (b) *Gr43a-GAL4/UAS-dTrpA1;Cha-GAL80/+* (n = 30, 32, 32)

Sup. Fig. 9. Cholinergic gustatory neurons are important in inducing sleep in starved flies.

Same as Sup. Fig 8 except for Gr-driver. (a) *Gr64a-GAL4/+;Cha-GAL80/+* (n = 32, 32, 32) (b) *Gr64a-GAL4/UAS-dTrpA1;Cha-GAL80/+* (n = 32, 31, 32)

Sup. Fig. 10. Inhibition of gustatory sugar receptor expressing neurons suppressed sweetener-induced sleep. Same as Fig. 4ab except for the difference of the Gr drivers. (a) *Gr5a-GAL4/+* (n = 19, 26, 25) (b) *Gr5a-GAL4/UAS-shi* (n = 32, 29, 30)

Sup. Fig. 11. Inhibition of gustatory sugar receptor expressing neurons suppressed sweetener-induced sleep. Same as Fig. 4ab except for the difference of the Gr drivers. (a) *Gr64a-GAL4/+* (n = 20, 24, 23) (d) *Gr64a-GAL4/UAS-shi* (n = 27, 30, 30)

a

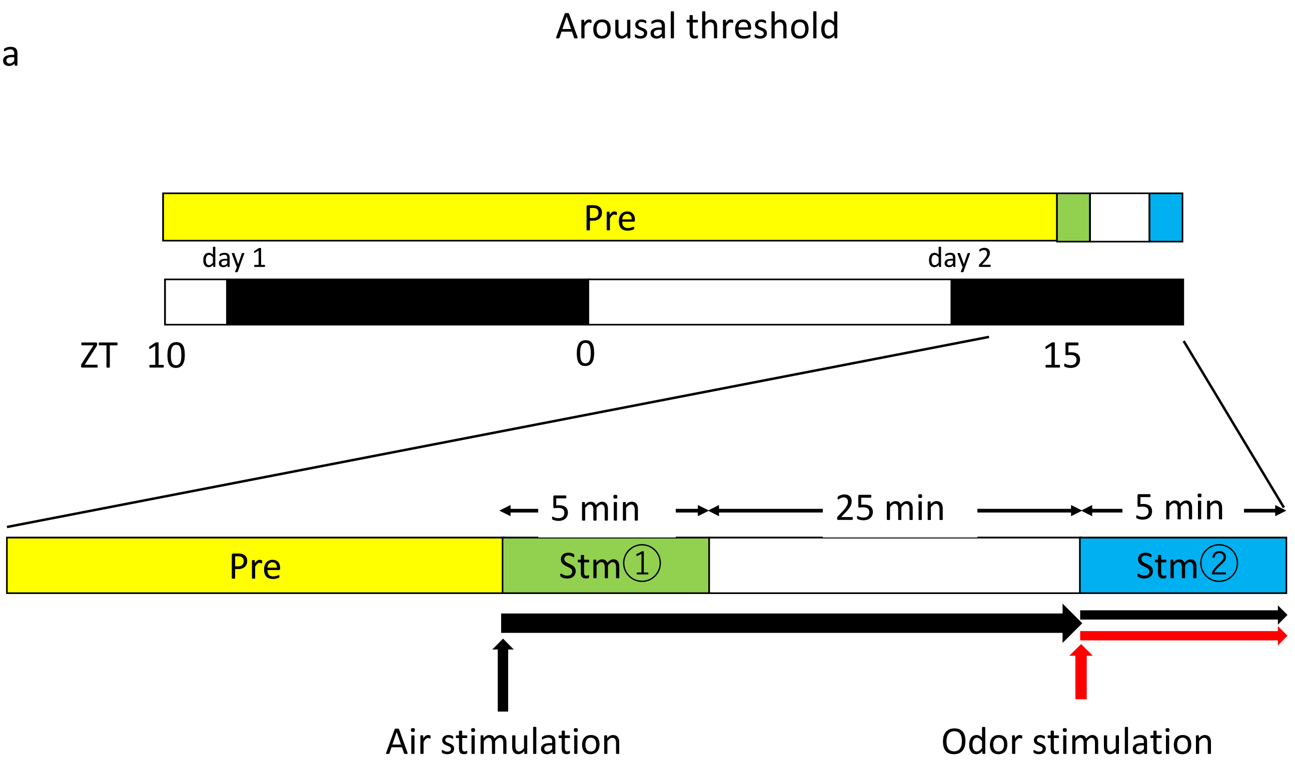
Arousal threshold
Pre
day 1
day 2
ZT 10
0
15
5 min
25 min
5 min
Pre
Stm①
Stm②
Air stimulation
Odor stimulation

b

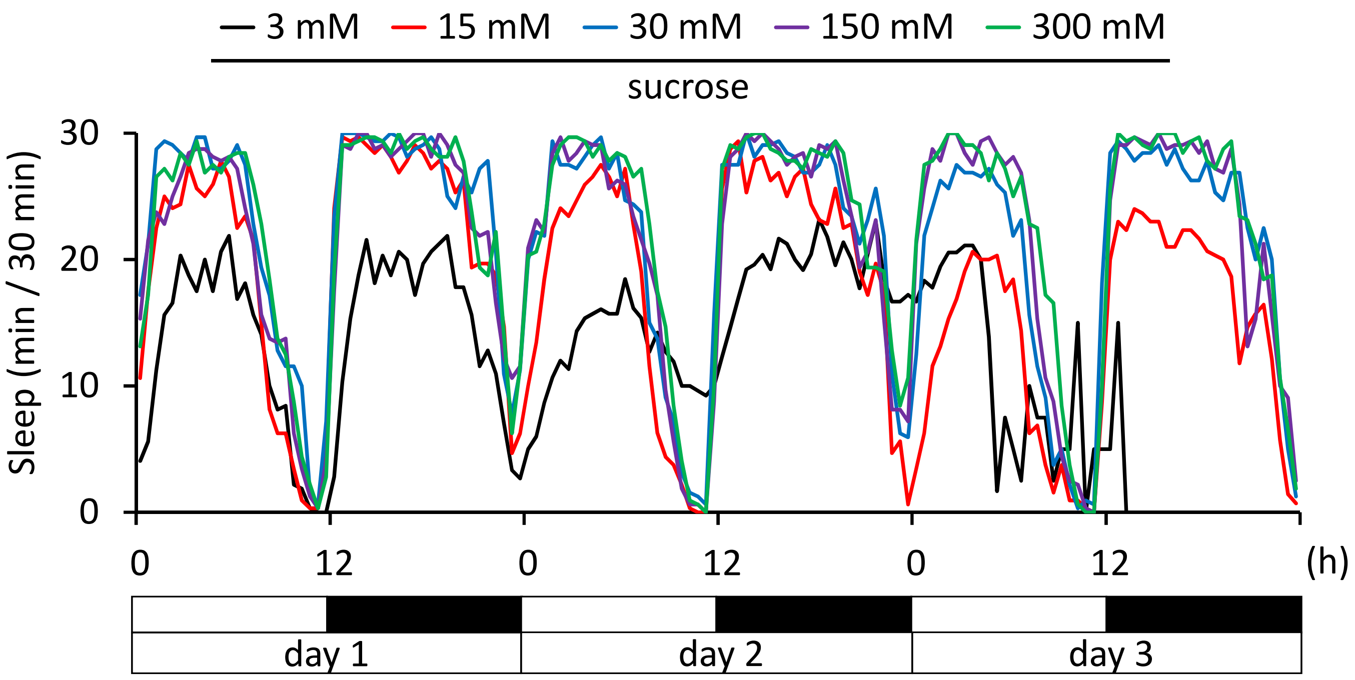
3 mM
15 mM
30 mM
150 mM
300 mM
sucrose
Sleep (min / 30 min)
30
20
10
0
0
12
0
12
0
12
(h)
day 1
day 2
day 3

Sup Fig 2

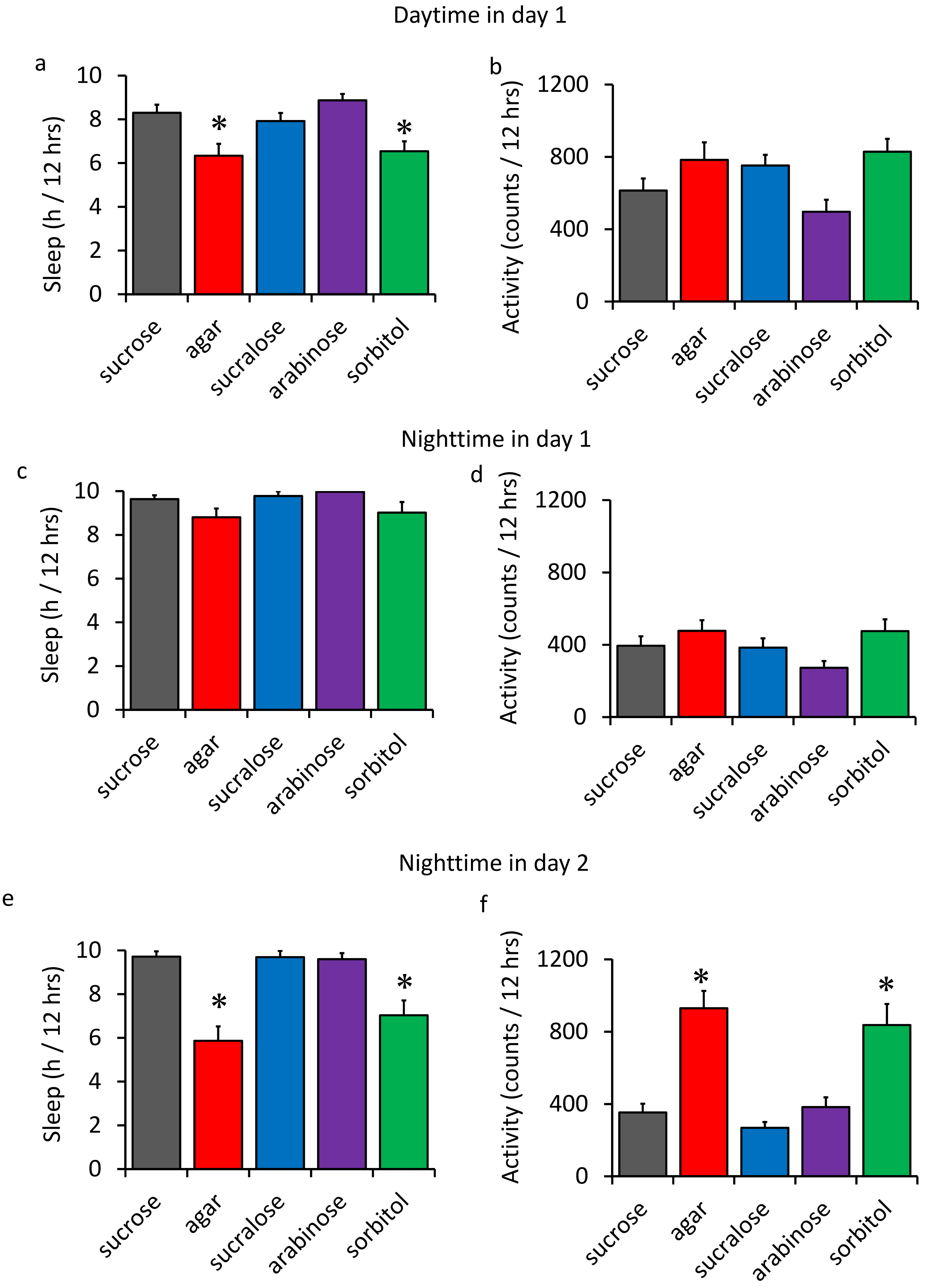
Daytime in day 1
a
Sleep (h / 12 hrs)
10
8
6
4
2
0
*
*
sucrose
agar
sucralose
arabinose
sorbitol
b
Activity (counts / 12 hrs)
1200
800
400
0
sucrose
agar
sucralose
arabinose
sorbitol
Nighttime in day 1
c
Sleep (h / 12 hrs)
10
8
6
4
2
0
sucrose
agar
sucralose
arabinose
sorbitol
d
Activity (counts / 12 hrs)
1200
800
400
0
sucrose
agar
sucralose
arabinose
sorbitol
Nighttime in day 2
e
Sleep (h / 12 hrs)
10
8
6
4
2
0
*
*
sucrose
agar
sucralose
arabinose
sorbitol
f
Activity (counts / 12 hrs)
1200
800
400
0
*
*
sucrose
agar
sucralose
arabinose
sorbitol

Sup Fig 3

a

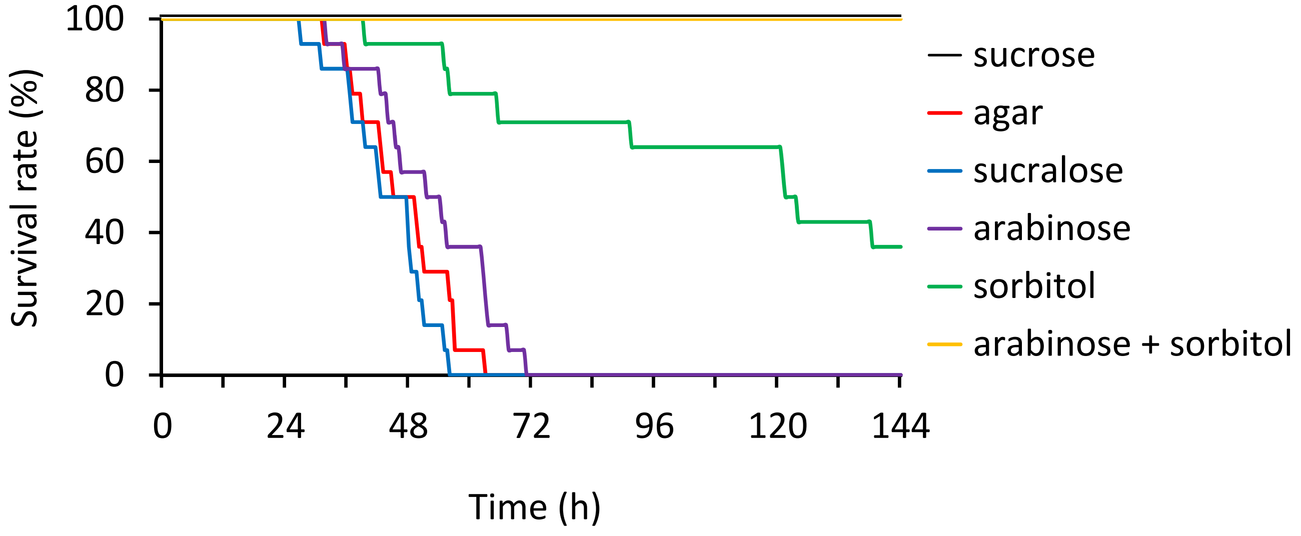
Survival rate (%)
100
80
60
40
20
0
0
24
48
72
96
120
144
Time (h)
sucrose
agar
sucralose
arabinose
sorbitol
arabinose + sorbitol

b

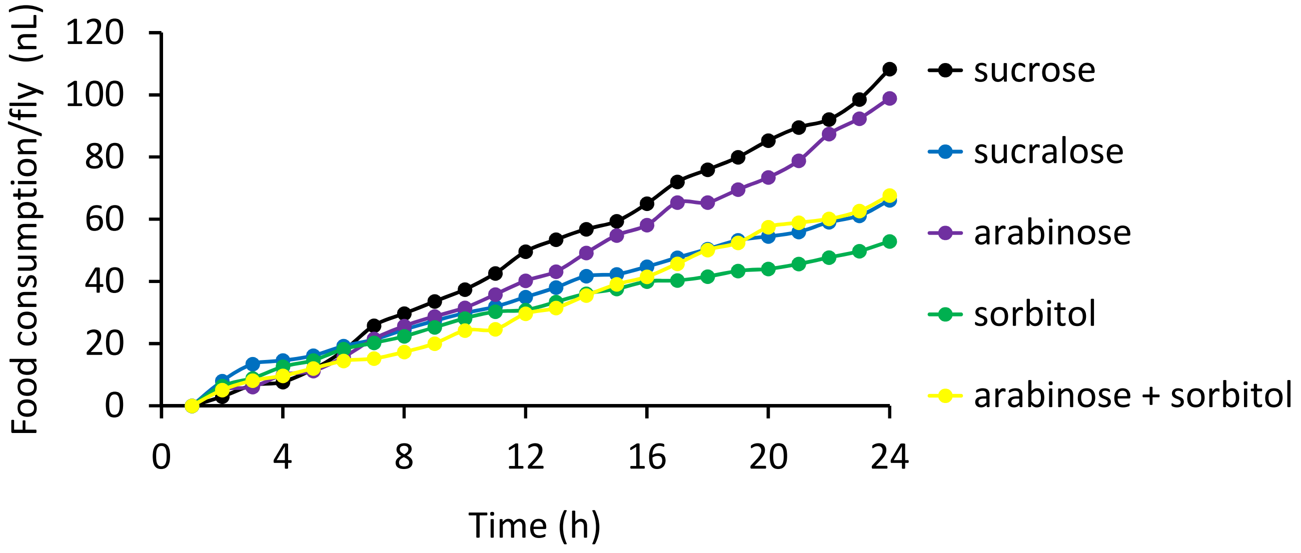
Food consumption/fly (nL)
120
100
80
60
40
20
0
0
4
8
12
16
20
24
Time (h)
sucrose
sucralose
arabinose
sorbitol
arabinose + sorbitol

Sup Fig 4

a

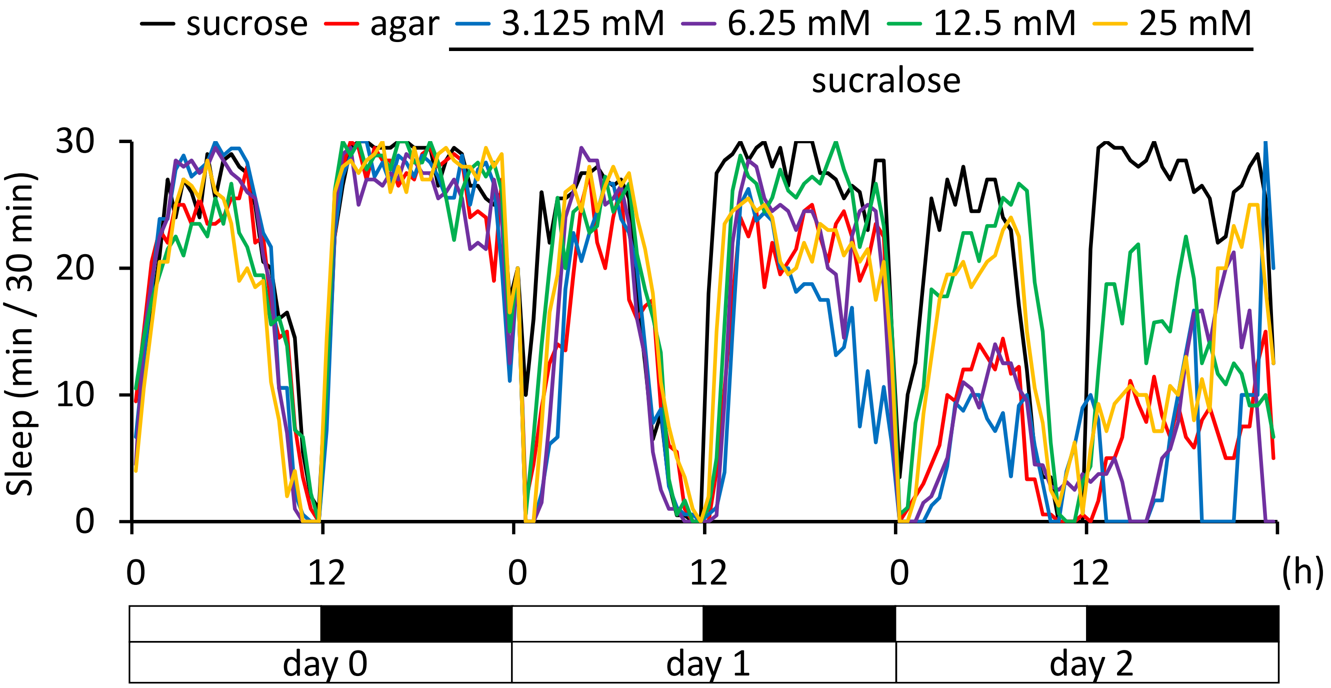

sucrose
agar
3.125 mM
6.25 mM
12.5 mM
25 mM
sucralose
Sleep (min / 30 min)
30
20
10
0
0
12
0
12
0
12
(h)
day 0
day 1
day 2

b

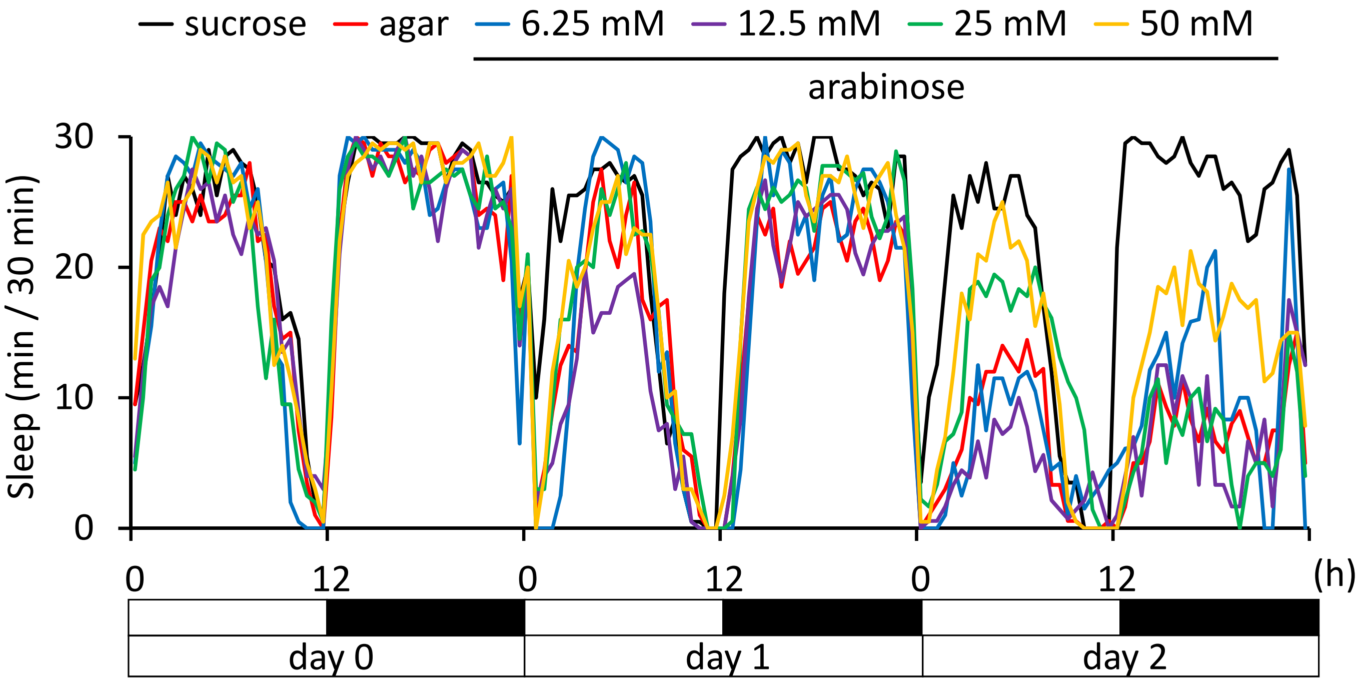

sucrose
agar
6.25 mM
12.5 mM
25 mM
50 mM
arabinose
Sleep (min / 30 min)
30
20
10
0
0
12
0
12
0
12
(h)
day 0
day 1
day 2

Sup Fig 5

a

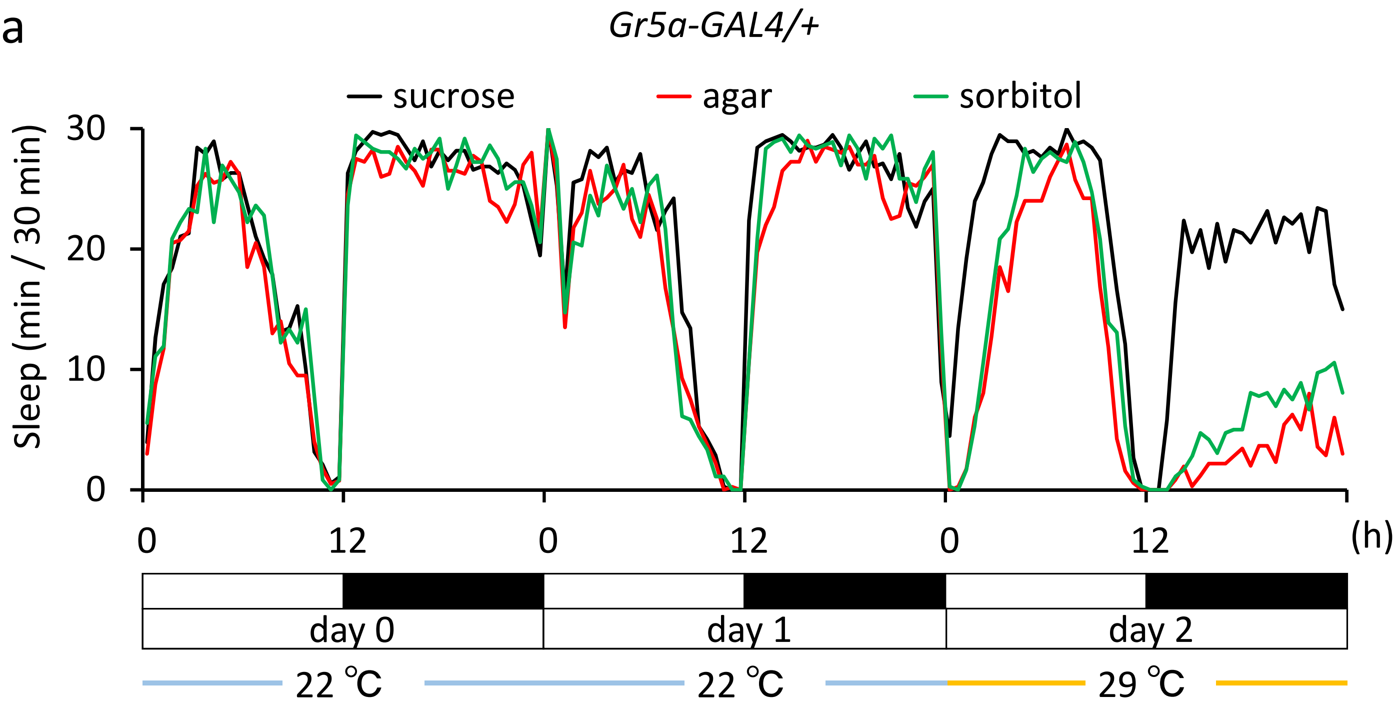
Gr5a-GAL4/+
sucrose
agar
sorbitol
Sleep (min / 30 min)
30
20
10
0
0
12
0
12
0
12
(h)
day 0
day 1
day 2
22 °C
22 °C
29 °C

b

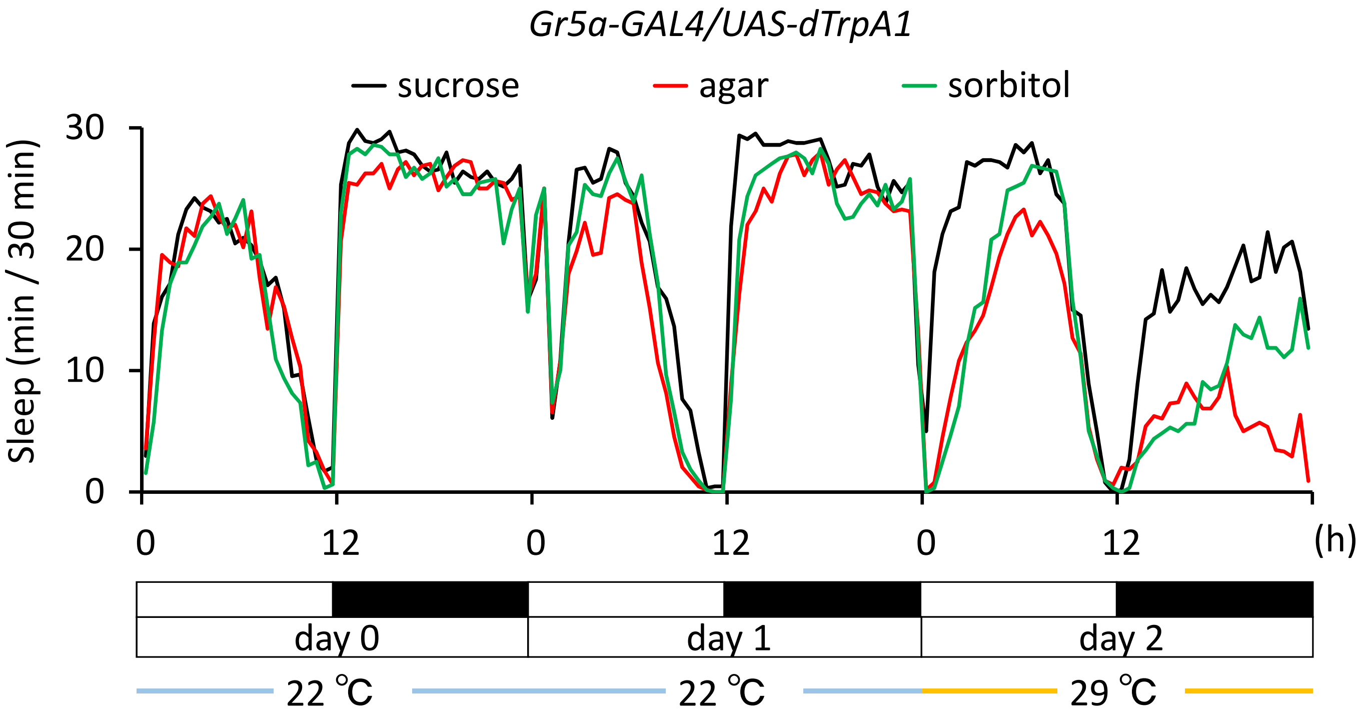
Gr5a-GAL4/UAS-dTrpA1
sucrose
agar
sorbitol
Sleep (min / 30 min)
30
20
10
0
0
12
0
12
0
12
(h)
day 0
day 1
day 2
22 °C
22 °C
29 °C

Sup Fig 6

a

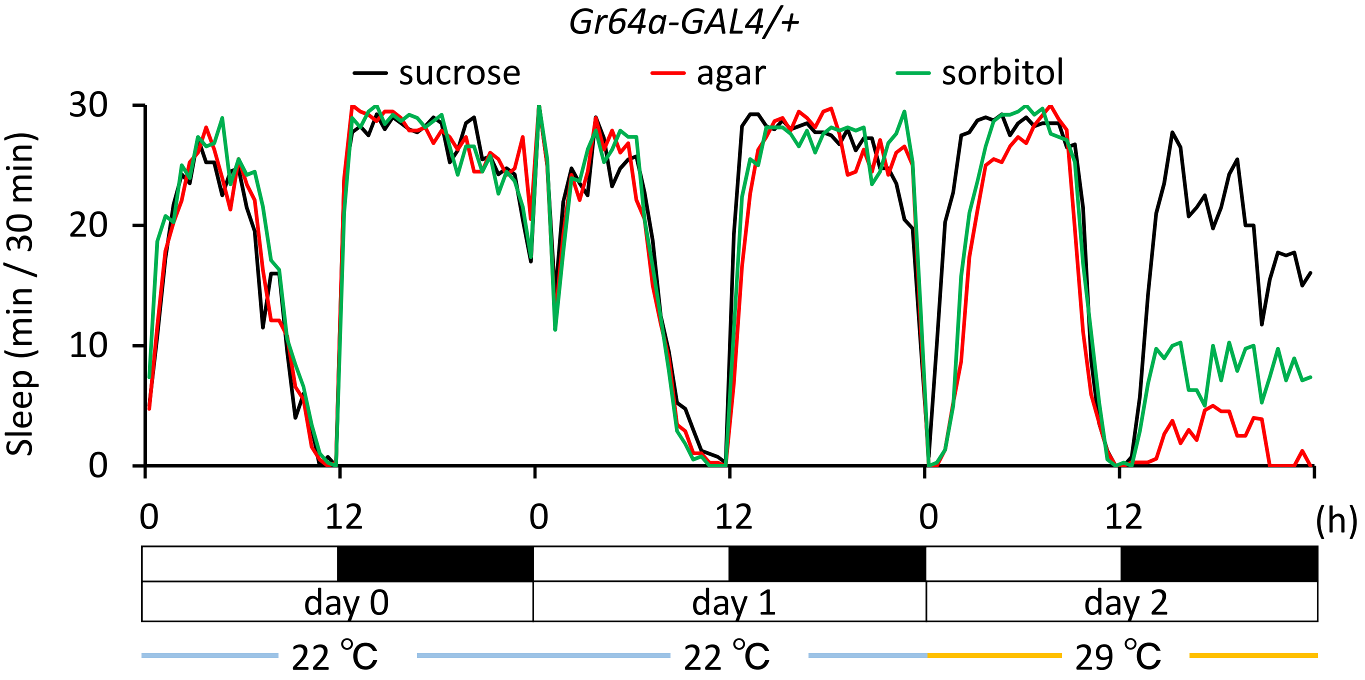
Gr64a-GAL4/+
sucrose
agar
sorbitol
Sleep (min / 30 min)
30
20
10
0
0
12
0
12
0
12
(h)
day 0
day 1
day 2
22 °C
22 °C
29 °C

b

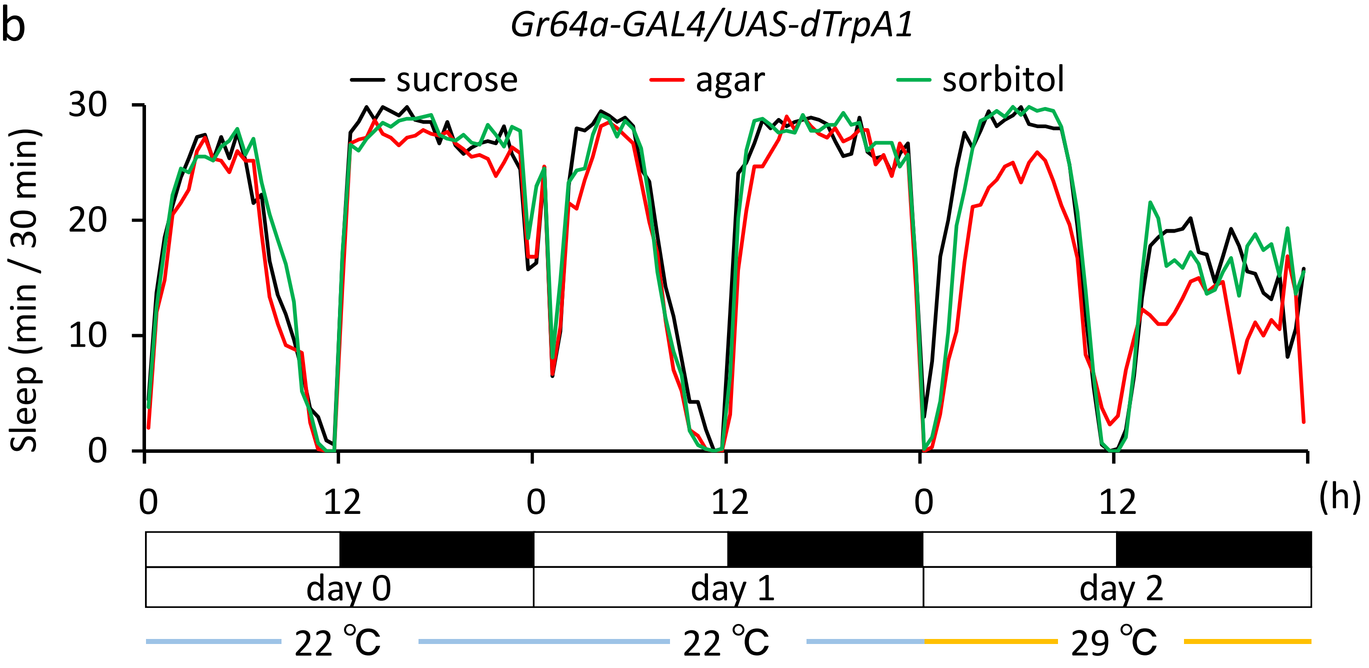
Gr64a-GAL4/UAS-dTrpA1
sucrose
agar
sorbitol
Sleep (min / 30 min)
30
20
10
0
0
12
0
12
0
12
(h)
day 0
day 1
day 2
22 °C
22 °C
29 °C

Sup Fig 7

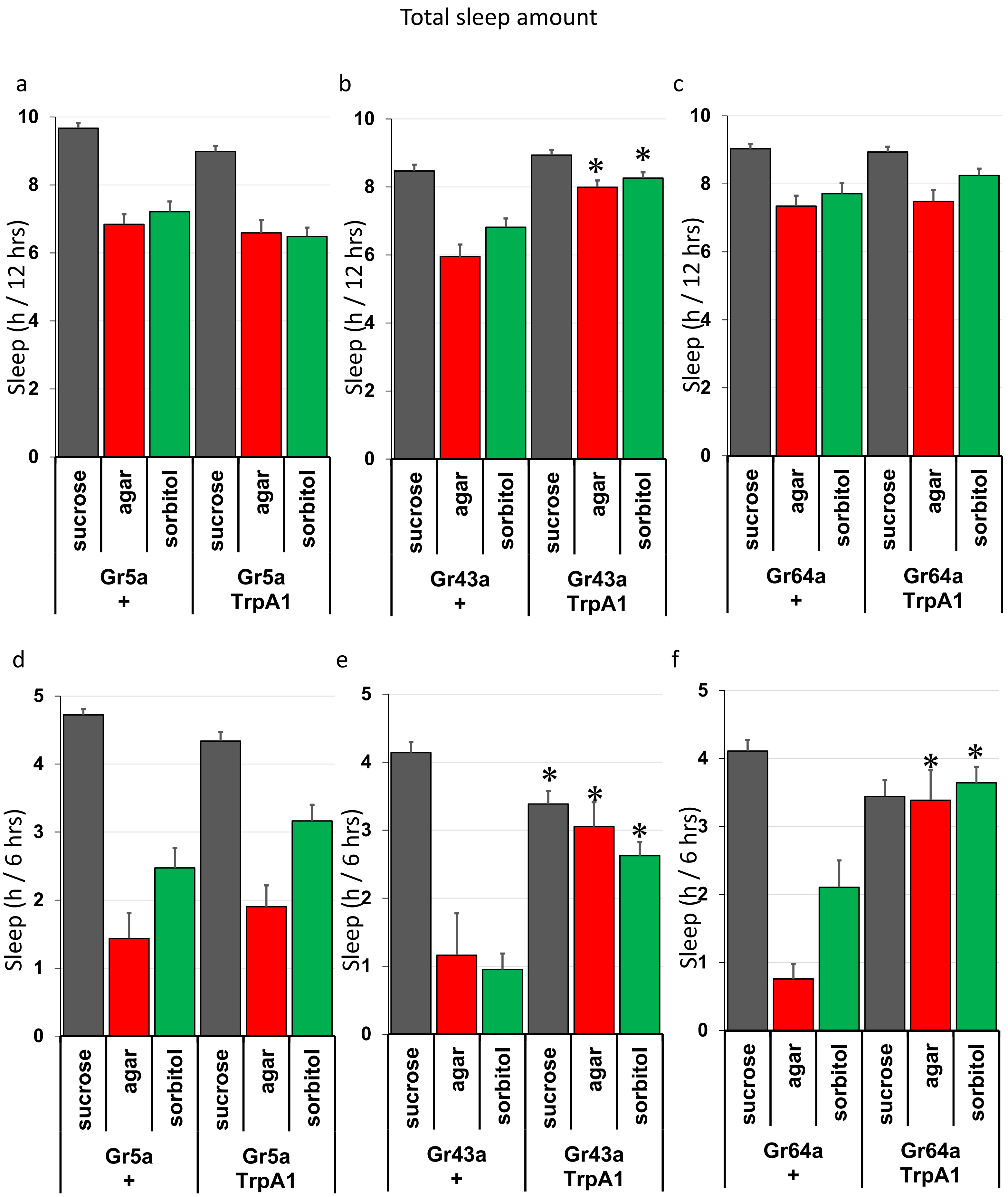
Total sleep amount
a
b
c
d
e
f
Sleep (h / 12 hrs)
Sleep (h / 6 hrs)
10
8
6
4
2
0
5
4
3
2
1
0
sucrose
agar
sorbitol
Gr5a
+
Gr5a
TrpA1
Gr43a
+
Gr43a
TrpA1
Gr64a
+
Gr64a
TrpA1
*

Sup Fig 8

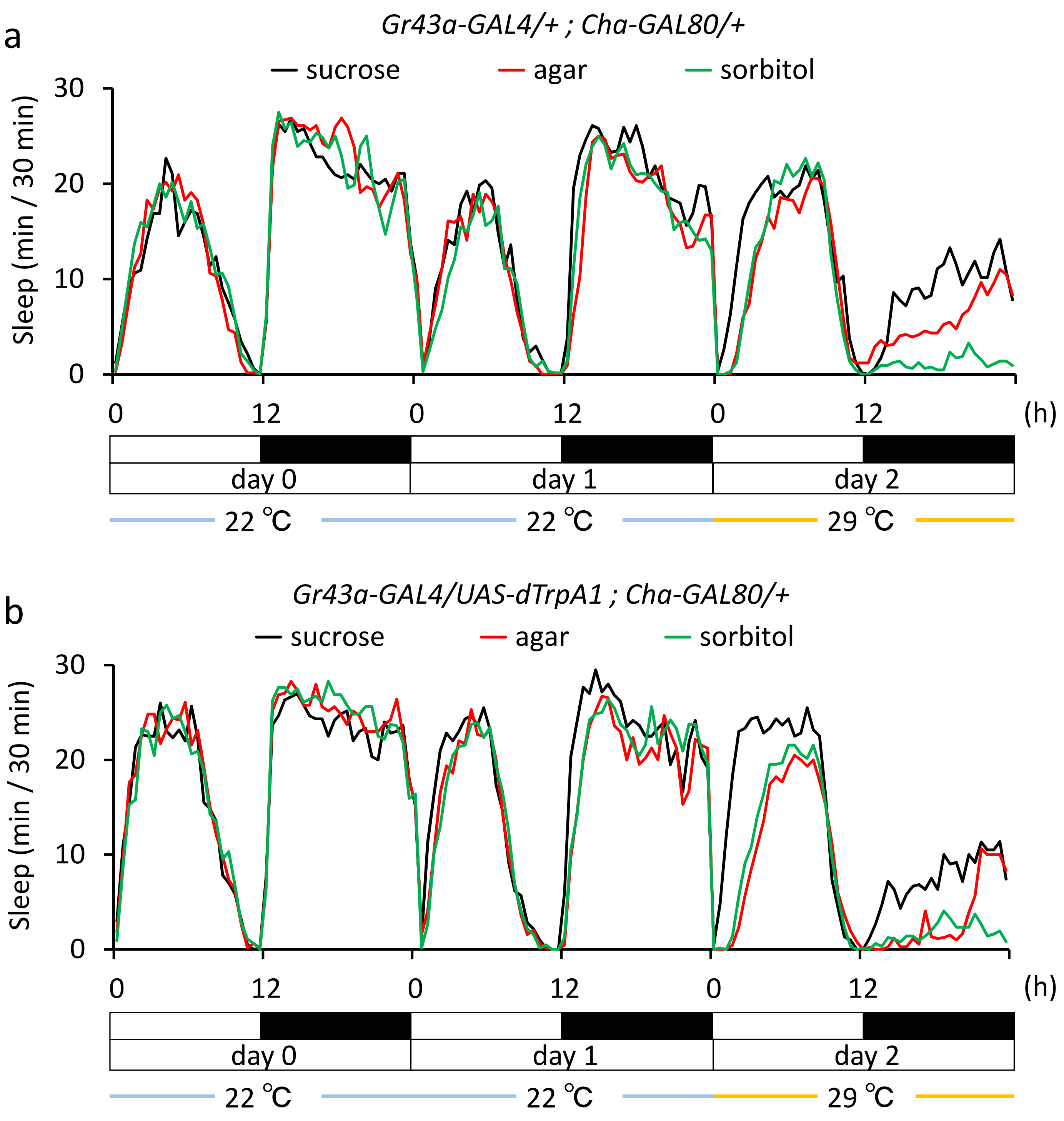
a
Gr43a-GAL4/+ ; Cha-GAL80/+
sucrose
agar
sorbitol
Sleep (min / 30 min)
30
20
10
0
0
12
0
12
0
12
(h)
day 0
day 1
day 2
22 °C
22 °C
29 °C
b
Gr43a-GAL4/UAS-dTrpA1 ; Cha-GAL80/+
sucrose
agar
sorbitol
Sleep (min / 30 min)
30
20
10
0
0
12
0
12
0
12
(h)
day 0
day 1
day 2
22 °C
22 °C
29 °C

Sup Fig 9

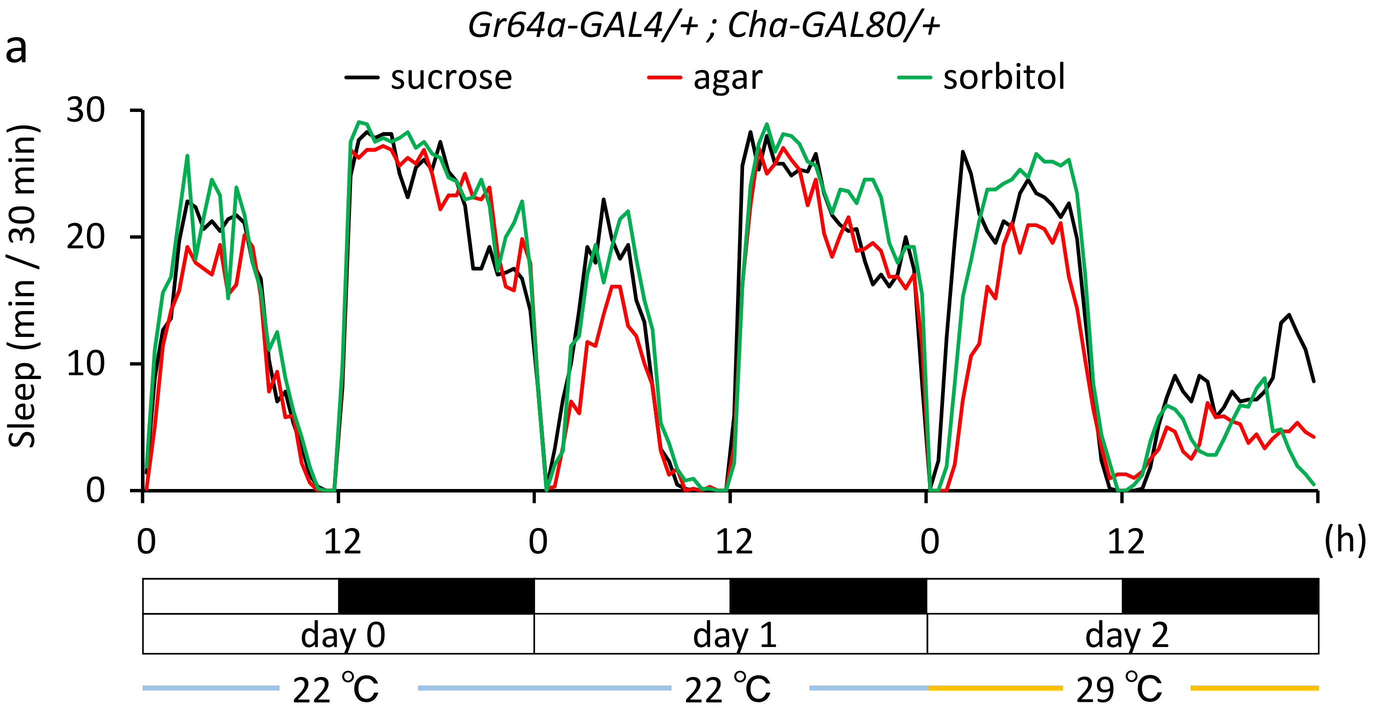

a
Gr64a-GAL4/+ ; Cha-GAL80/+
sucrose
agar
sorbitol
Sleep (min / 30 min)
30
20
10
0
0
12
0
12
0
12
(h)
day 0
day 1
day 2
22 °C
22 °C
29 °C

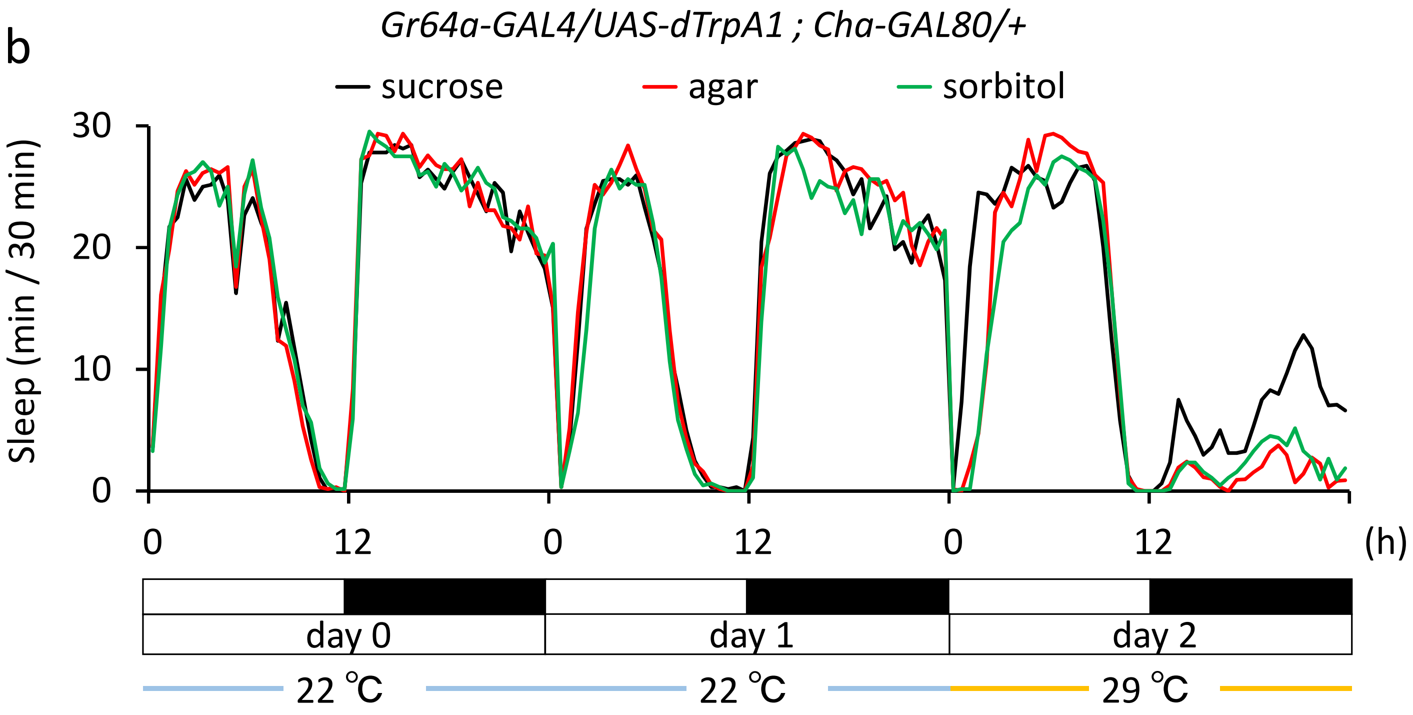

b
Gr64a-GAL4/UAS-dTrpA1 ; Cha-GAL80/+
sucrose
agar
sorbitol
Sleep (min / 30 min)
30
20
10
0
0
12
0
12
0
12
(h)
day 0
day 1
day 2
22 °C
22 °C
29 °C

Sup Fig 10

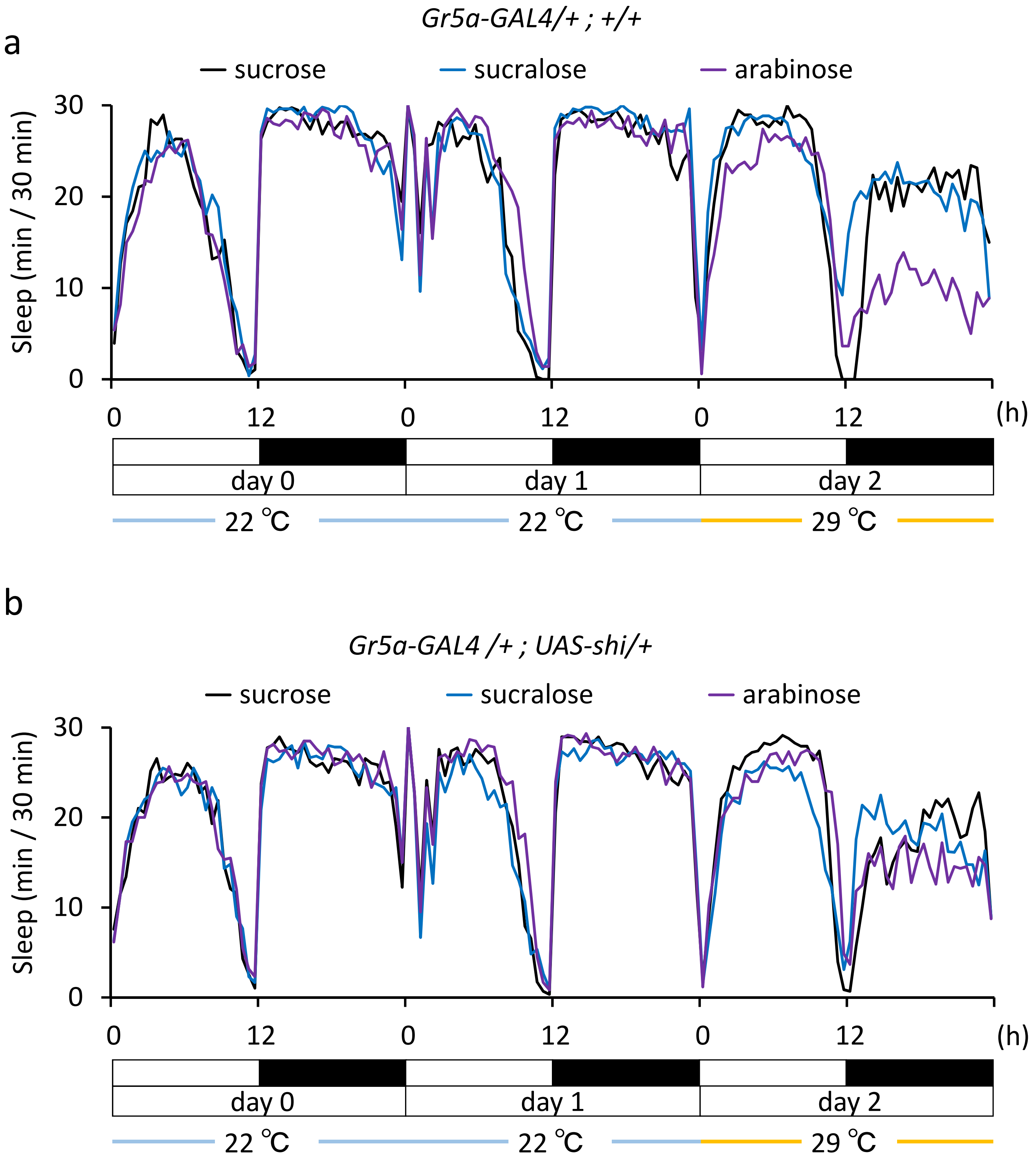
a
Gr5a-GAL4/+ ; +/+
sucrose
sucralose
arabinose
Sleep (min / 30 min)
30
20
10
0
0
12
0
12
0
12
(h)
day 0
day 1
day 2
22 °C
22 °C
29 °C
b
Gr5a-GAL4 /+ ; UAS-shi/+
sucrose
sucralose
arabinose
Sleep (min / 30 min)
30
20
10
0
0
12
0
12
0
12
(h)
day 0
day 1
day 2
22 °C
22 °C
29 °C

Sup Fig 11

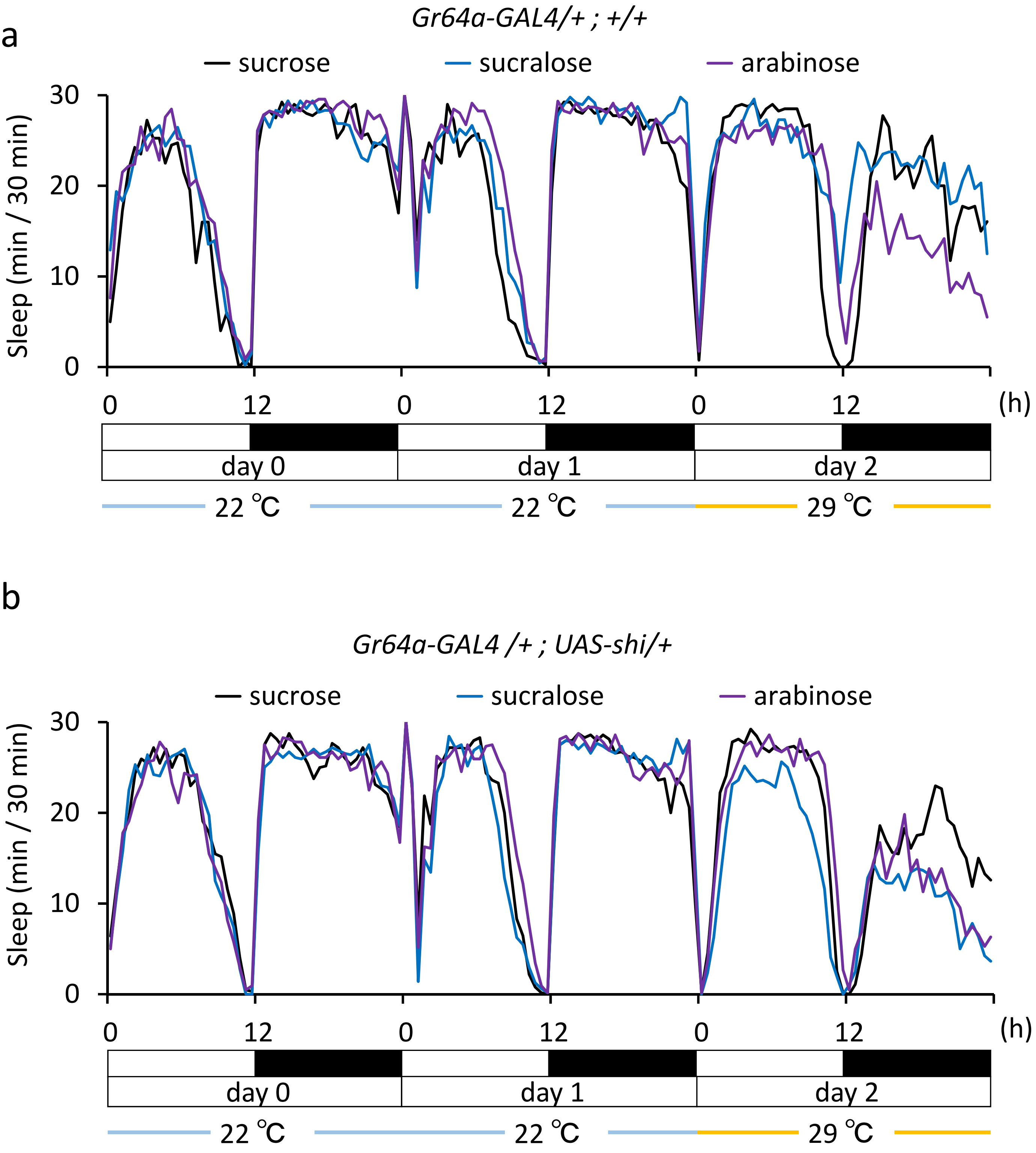
a
Gr64a-GAL4/+ ; +/+
sucrose
sucralose
arabinose
Sleep (min / 30 min)
30
20
10
0
0
12
0
12
0
12
(h)
day 0
day 1
day 2
22 °C
22 °C
29 °C
b
Gr64a-GAL4 /+ ; UAS-shi/+
sucrose
sucralose
arabinose
Sleep (min / 30 min)
30
20
10
0
0
12
0
12
0
12
(h)
day 0
day 1
day 2
22 °C
22 °C
29 °C
